# Global trends and risk factors of chronic kidney disease in children and young adults from 1990 to 2021: a systematic analysis of the global burden of disease study 2021

**DOI:** 10.3389/fpubh.2025.1696021

**Published:** 2025-12-18

**Authors:** Yapeng He, Yan Qian, Qiuting Xu, Qunfeng Lu, Nina Zhang

**Affiliations:** Department of Nursing, Shanghai Sixth People’s Hospital Affiliated to Shanghai Jiao Tong University School of Medicine, Shanghai, China

**Keywords:** CKD, GBD, children, young adults, epidemiology

## Abstract

**Introduction:**

Chronic kidney disease (CKD) is an important contributor to morbidity and mortality from non-communicable disease in children and young adults, but there is a lack of data on incidence, mortality, disability-adjusted life years (DALYs), risk factors, and trends in this population. This study aims to provide comprehensive estimates of the burden of CKD among children and young adults.

**Methods:**

We conducted a trend analysis using data from the Global Burden of Diseases, Injuries, and Risk Factors Study (GBD) 2021. Measures of burden at the global, sociodemographic index (SDI), and regional levels for children and young adults included incidence, mortality, and DALY rates per 100,000 population due to CKD, as well as attributable risks of death and DALYs, the annual percentage change (APC), and the average annual percentage change (AAPC). We also investigated the association between CKD burden and SDI and predicted the incidence from 2022 to 2050.

**Results:**

Globally, from 1990 to 2021, children with CKD demonstrated declining trends in mortality and DALYs, with AAPCs of −1.925 and −1.820, respectively; however, the incidence rate did not change significantly, although it showed a negative trend overall (AAPCs: −0.095). Conversely, all three metrics showed upward trajectories in young adults, with AAPCs of 0.941, 0.256, and 0.187. The relationship between incidence and SDI exhibited an inverse U-shaped pattern, while also demonstrating significant negative associations with mortality and DALYs. Notably, region-specific disparities emerged in attributable risk factors for mortality and DALYs among young adults. Higher SDI regions displayed greater proportional contributions from high fasting plasma glucose, high body mass index, and diets high in processed meat and red meat. Projections for 2022–2050 suggest a continued reduction in children’s CKD incidence, while projections for young adults’ incidence suggest a continued increase.

**Conclusion:**

The global burden of CKD shows divergent trends across age groups, with projected increases among young adults contrasting with projected declines among children. Lower SDI regions demonstrate a disproportionately higher burden of CKD.

## Introduction

Chronic kidney disease (CKD) is a major worldwide clinical and public health problem. According to the Kidney Disease: Improving Global Outcomes (KDIGO) 2012 guidelines, CKD is defined as abnormalities of kidney structure or function, present for > 3 months, with health implications, and the guidelines recommend that CKD can be classified based on glomerular filtration rate (GFR) category and albuminuria category. The diagnostic thresholds for CKD are an estimated glomerular filtration rate (eGFR) of less than 60 mL/min/1.73 m^2^ and an albumin–creatinine ratio (ACR) of 30 mg/g or more (1, 2). In 2017, approximately 850 million people worldwide were affected by some form of CKD, resulting in 1.2 million deaths (3). By 2040, CKD is estimated to become the fifth leading cause of death (4).

Globally, CKD due to diabetes and hypertension accounts for 50.62 and 23.26% of the overall increase in the burden of CKD, respectively (5). At the same time, the prevalence of confirmed hypertension remains at 2–4% in children, and this prevalence continues to increase, indicating a younger trend of hypertension (6, 7). Type 2 diabetes mellitus (T2DM) was traditionally recognized as a disease of the middle-aged and older adult population, but since the 2000s, the greatest relative increase in the incidence and prevalence of T2DM has been observed in young adults, adolescents, and even children (8). A 2013 systematic review reported that the prevalence of T2DM ranged from 0 to 5,300 cases per 100,000 adolescents (9). In addition to traditional risk factors for late-onset T2DM, family history, genetics, and socioeconomic status may play a key role in young-onset T2DM (10). The standardized mortality ratio for young-onset T2DM was twice as high as that for late-onset T2DM (11). Furthermore, children with kidney failure have up to 32 years of life lost, and those who develop kidney failure at the youngest ages lose the most life years (12). It has been found that the population of children and young adults with CKD represents a significant and often overlooked subpopulation within the broader CKD population (13). Therefore, this study aims to describe the incidence, mortality, disability-adjusted life years (DALYs), and risk factors due to CKD in children and young adults at global and regional levels, from 1990 to 2021, using data from the Global Burden of Diseases, Injuries, and Risk Factors Study (GBD) 2021.

## Methods

### Study design and data sources

The GBD 2021 was a comprehensive multinational effort to estimate the burden of disease, drawing data from 204 countries or territories.^1^ The present study focused on extracting data related to CKD incidence, mortality, DALYs, risk factors, and percentage change from 1990 to 2021 in children (0–14 years) and young adults (15–39 years), with significant differences in terms of sex, country, region, and sociodemographic index (SDI). In addition, we extended the GBD forecasts of CKD incidence by extracting global population estimates for 1990–2021^2^ and global population forecasts for 2022–2050.^3^ Given that this study did not use identifiable data, did not involve participants in the data collection process or outcome measures, and did not require ethics approval or informed consent, the requirements for ethics approval and informed consent were deemed unnecessary and impractical for this study.

### Measures of burden

Measures of burden at global, SDI, and regional levels included incidence, mortality, DALYs, percent change, and related 95% uncertainty intervals (UIs) for CKD, stratified by sex. Methods for processing, standardization, and modeling of CKD incidence and mortality have been introduced by the GBD Chronic Kidney Disease Collaboration (14). In brief, the incidence of CKD was estimated using a wide range of data from the representative population, and mortality was estimated by modelling mortality assigned to CKD diagnostic codes using vital registration, verbal autopsy, and surveillance system data for 1990–2021. DALYs represent the total health burden attributable to disease and were calculated as the sum of years of life lost (YLLs) and years lived with disability (YLDs). The attributable risks of death and DALYs were categorized into three levels: combined environmental or occupational risks; behavioral risks; and metabolic risks, such as low temperature, high systolic blood pressure, or diets high in sodium. All measures are reported as raw values, percentages, and age-standardized rates (ASRs) per 100,000 people.

### Country classification

In this study, we classified all countries into 21 geographic regions according to the GBD region framework and used the 2021 SDI, divided into quintiles ranging from 0 (worst) to 100 (best), to define high (~100), high-middle (~80), middle (~60), low-middle (~40), and low (~20) (15). SDI is a composite measure of lag-distributed income per capita, mean years of education, and total fertility under 25 years of age. A higher SDI indicates better socioeconomic conditions (15). Each geographic region and SDI group was listed in Table 1.

**Table 1:** Age-standardized incidence rate for chronic kidney disease in children and young adults from 1990 to 2021.

Characteristics	Age-standardized incidence rate per 100,000 population in 1990 (95% UI)		Age-standardized incidence rate per 100,000 population in 2021 (95% UI)		Percentage change from 1990 to 2021, % (95% UI)	
	0–14 years	15–39 years	0–14 years	15–39 years	0–14 years	15–39 years
Global	31.6 (27.0–37.4)	27.0 (20.4–34.6)	30.6 (26.4–35.9)	36.1 (28.3–45.2)	−3.3 (−8.4–2.3)	33.6 (23.0–47.1)^*^
Male	29.5 (25.3–34.6)	25.1 (19.1–32.0)	28.9 (24.9–33.9)	34.6 (27.5–42.9)	−2.0 (−7.6–4.5)	38.1 (27.7–52.1)^*^
Female	33.9 (28.7–40.1)	28.9 (21.7–37.3)	32.4 (28.0–38.0)	37.5 (29.2–47.5)	−4.3 (−9.1–0.9)	29.6 (19.0–43.4)^*^
SDI groups						
High SDI	28.0 (23.1–33.5)	19.2 (13.2–26.2)	26.5 (22.4–31.4)	23.3 (16.5–30.8)	−5.5 (−10.3 - -0.4)^*^	21.8 (11.1–37.7)^*^
High-middle SDI	30.5 (25.7–36.4)	25.3 (18.8–33.0)	25.5 (21.3–30.8)	33.6 (24.8–43.7)	−16.5 (−20.2 - -12.5)^*^	32.7 (22.5–45.1)^*^
Middle SDI	34.1 (29.1–40.4)	29.8 (22.9–37.9)	32.5 (27.9–38.6)	42.9 (34.3–53.5)	−4.8 (−10.1–1.3)	44.2 (30.3–62.2)^*^
Low-middle SDI	30.8 (26.4–36.5)	31.1 (24.2–39.3)	32.0 (27.4–37.4)	38.9 (31.3–48.3)	3.6 (−1.9–9.8)	25.3 (16.7–36.2)^*^
Low SDI	31.2 (27.0–36.1)	24.3 (18.8–30.3)	30.7 (27.0–35.2)	29.0 (23.6–35.6)	−1.8 (−7.7–5.1)	19.5 (8.7–31.2)^*^
Geographic regions						
East Asia	29.9 (25.6–35.3)	21.6 (16.0–28.4)	19.2 (15.6–23.8)	25.0 (17.9–32.5)	−36.0 (−41.0 - -30.2)^*^	15.5 (2.1–30.9)^*^
Oceania	33.7 (28.0–40.0)	40.6 (32.3–50.0)	36.3 (30.9–42.6)	47.7 (38.7–57.4)	7.8 (−1.8–18.5)	17.6 (4.0–32.4)^*^
Southeast Asia	35.7 (30.5–42.1)	32.7 (25.3–41.8)	39.3 (33.9–45.7)	46.7 (37.8–56.8)	10.2 (3.6–18.5)^*^	42.7 (28.4–62.1)^*^
Central Asia	43.8 (36.5–53.1)	50.4 (39.0–63.2)	48.6 (40.6–57.8)	67.8 (53.6–82.7)	11.0 (4.7–18.6)^*^	34.4 (22.6–48.2)^*^
Central Europe	25.1 (20.6–30.3)	25.4 (17.6–34.0)	23.7 (19.6–28.7)	33.9 (25.4–44.6)	−5.6 (−10.4 - -1.0)^*^	33.6 (23.5–49.3)^*^
Eastern Europe	44.6 (37.2–53.6)	40.3 (29.5–52.8)	39.3 (32.2–48.5)	64.6 (47.6–83.6)	−11.8 (−16.6 - -6.6)^*^	60.5 (47.9–76.6)^*^
Australasia	18.6 (14.7–22.8)	11.8 (7.7–17.4)	19.6 (15.8–24.5)	16.9 (10.9–23.9)	5.3 (−4.6–19.5)	43.0 (21.9–70.8)^*^
High-Income Asia Pacific	19.4 (15.3–24.2)	22.3 (15.3–30.1)	17.1 (13.3–21.7)	21.7 (15.0 29.6)	−11.9 (−16.6 - -7.3)^*^	−2.6 (−9.1–4.2)
High-Income North America	40.6 (33.6–48.8)	20.5 (13.5–28.8)	35.7 (30.7–41.4)	17.8 (11.7–25.1)	−12.0 (−19.4 - -4.7)^*^	−12.8 (−25.2–3.4)
Southern Latin America	27.0 (22.2–32.0)	14.4 (9.8–20.0)	25.8 (22.0–30.2)	17.7 (12.2–24.3)	−4.4 (−13.2–6.9)	22.4 (−1.2–51.6)
Western Europe	17.2 (13.7–21.4)	11.2 (7.2–16.3)	15.4 (12.2–18.9)	12.0 (7.9–17.4)	−10.9 (−14.7 - -6.2)^*^	7.1 (−2.4–18.7)
Andean Latin America	29.8 (25.2–35.6)	22.3 (16.4–29.2)	37.7 (32.2–42.2)	35.6 (27.3–45.9)	26.5 (14.5–42.6)^*^	59.9 (37.0–91.1)^*^
Caribbean	32.9 (27.5–39.6)	34.8 (26.7–44.4)	37.7 (32.1–44.7)	55.9 (45.1–69.0)	14.6 (4.4–25.8)^*^	60.6 (42.0–85.1)^*^
Central Latin America	41.4 (34.2–49.7)	40.2 (29.9–51.8)	49.9 (43.1–58.5)	70.2 (58.8–83.9)	20.5 (9.2–34.4)^*^	74.5 (50.1–109.0)^*^
Tropical Latin America	28.8 (24.2–34.6)	32.3 (24.6–41.8)	30.8 (26.5–36.2)	41.8 (31.8–52.3)	7.0 (1.6–12.5)^*^	29.4 (15.2–47.8)^*^
North Africa and the Middle East	35.3 (30.0–41.7)	31.8 (24.4–40.8)	40.5 (34.8–47.9)	56.2 (43.7–71.0)	14.7 (7.8–22.7)^*^	76.6 (56.9–98.4)^*^
South Asia	28.7 (24.3–34.2)	32.8 (25.7–41.5)	27.0 (22.6–32.3)	36.4 (29.1–45.3)	−5.9 (−11.5–0.5)	11.0 (4.1–21.3)^*^
Central sub-Saharan Africa	28.8 (24.9–33.3)	17.1 (12.9–21.6)	26.8 (22.9–31.3)	22.2 (17.4–27.7)	−6.7 (−15.9–2.9)	29.6 (9.9–51.4)^*^
Eastern sub-Saharan Africa	27.3 (23.7–31.4)	16.0 (11.9–20.5)	24.7 (21.8–28.0)	18.0 (14.0–22.6)	−9.7 (−16.4 - -2.7)^*^	12.8 (−1.5–29.8)
Southern sub-Saharan Africa	28.7 (23.8–34.6)	36.7 (28.6–46.2)	26.8 (22.7–32.1)	45.0 (36.1–54.8)	−6.7 (−12.2 - -1.0)^*^	22.6 (10.3–38.1)^*^
Western sub-Saharan Africa	42.1 (36.9–48.2)	29.0 (22.1–36.6)	39.4 (34.8–45.1)	35.7 (28.6–43.5)	−6.4 (−11.2 - -0.9)^*^	23.2 (13.0–35.9)^*^

^*^Percentage change is significant; UI, uncertainty interval; SDI, sociodemographic index.

### Joinpoint regression model

The joinpoint regression model was first proposed by Kim et al. (16). This type of non-linear regression model is composed of a few continuous linear phases and is often useful for describing trends in data. The equation of the log-linear model is: *E[y/x]* = *e^β0 + β1x + δ1(x − τ1)+ +. + δk(x − τk)+^*, where *y* is incidence or mortality, *x* is year, *β_1_* is the regression coefficient, *k* is the number of joinpoints, the *τ_k_* are the unknown joinpoints, and *a^+^ = a* for *a* > 0 and 0 otherwise. The model also calculates the annual percentage change (APC) and average annual percentage change (AAPC) along with a confidence interval. The APC represents the trend of the segment, while the AAPC represents the average.

### Statistical analysis

We analyzed the age-standardized incidence, mortality, and DALYs of CKD in children and young adults in 1990 and 2021, respectively, to gain a preliminary understanding of the burden of CKD. We then used the ASRs of incidence, mortality, and DALYs for comparisons between different groups stratified by sex, SDI, and geographic regions. We calculated the percentage changes in the ASRs of incidence, mortality, and DALYs from 1990 to 2021 and depicted the burden changes at a global level and at different SDI levels. To further analyze the dynamic trend, we calculated the APC and AAPC using the joinpoint regression model. The correlations between SDI levels and age-standardized incidence, mortality, and DALYs were measured using Spearman’s rank-sum correlation tests. We also analyzed the risk factors of death and DALYs in CKD children and young adults and predicted the incidence from 2022 to 2050 to assess the future GBD of CKD. This GBD forecast is primarily based on the Global Population Forecasts 1990–2050 data and age-standardized CKD incidence data from 1990 to 2021. In our study, all statistical analyses were performed using the R program (version 4.4.2). A threshold of *p* value < 0.05 was regarded as a significant difference.

## Results

### Global burden and trends of CKD

**Figure 1:**
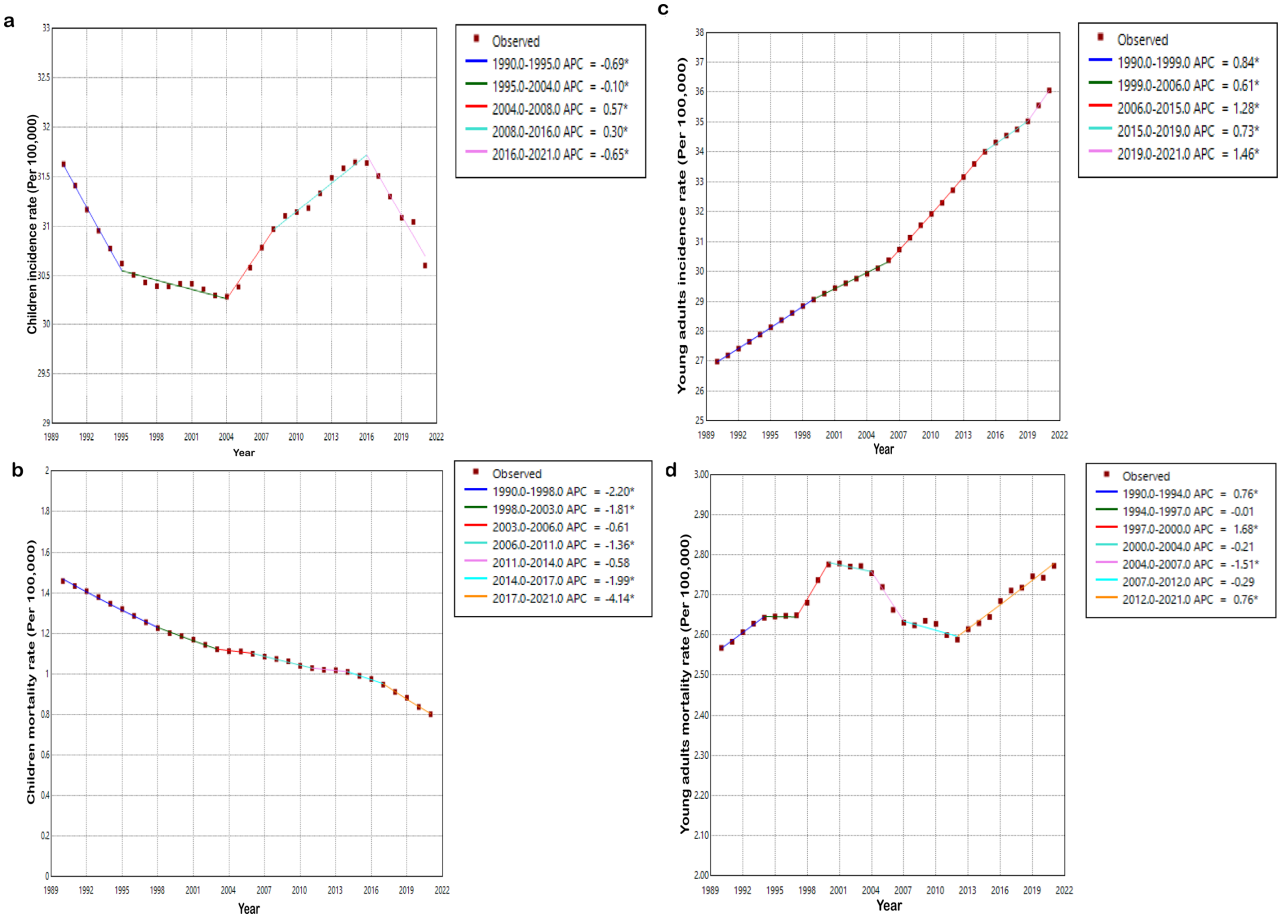
Global trends of CKD incidence and mortality rate in children and young adults during 1990-2021. **(a)** Trends in CKD incidence among children; **(b)** trends in CKD mortality among children; **(c)** trends in CKD incidence among young adults; **(d)** trends in CKD mortality among young adults.

Globally, the incidence of CKD in children declined from 31.6 (27.0–37.4) cases per 100,000 population in 1990 to 30.6 (26.4–35.9) cases per 100,000 population in 2021. Nevertheless, the percentage change was not statistically significant. Mortality decreased by 45.1%, from 1.5 (1.0–1.7) cases per 100,000 population to 0.8 (0.6–0.9) cases per 100,000 population. DALYs diminished by 43.2%, from 132.6 (96.1–151.1) cases per 100,000 population to 75.3 (60.8–87.2) cases per 100,000 population (Table 1, Supplementary Tables S1, S2). Furthermore, Figure 1 depicts the global trend in the burden of CKD. From 1990 to 2021, the AAPC of CKD incidence in children was −0.095 (from −0.139 to −0.051). The incidence demonstrated a significant downward trend during the periods 1990–1995 and 2016–2021, with the APC values being −0.687 and −0.653, respectively. The AAPC of mortality was −1.925 (from −2.129 to −1.720), and it showed a significant decline during 1990–1998 and 2017–2021, with the APC values of −2.196 and −4.138, respectively. The AAPC of DALYs was −1.820 (from −2.002 to −1.638), and it also presented a significant decrease during 1990–1998 and 2017–2021, with APC values of −2.117 and −3.779, respectively (Supplementary Table S3).

Among young adults, the incidence of CKD increased by 33.6%, rising from 27.0 (20.4–34.6) cases per 100,000 population in 1990 to 36.1 (28.3–45.2) cases per 100,000 population in 2021. The mortality rate increased from 2.6 (2.3–2.9) cases per 100,000 population to 2.8 (2.5–3.1) cases per 100,000 population, and DALYs increased from 198.3 (176.8–223.9) cases per 100,000 population to 211.1 (188.7–236.7) cases per 100,000 population. However, the percentage change was not statistically significant. In terms of the global trend from 1990 to 2021, the AAPC of CKD incidence was 0.941 (0.927–0.955), with the most rapid growth rate occurring during 2019–2021, and the APC was 1.458. The AAPC for mortality was 0.256 (0.035–0.477). It displayed significant growth during 1997–2000 and a significant decline during 2004–2007, with APC values of 1.681 and −1.514, respectively. The AAPC of DALYs was 0.187 (0.005–0.368), with significant growth during 1997–2000 (APC = 1.283) and a significant decline during 2004–2007 (APC = −1.223) (Supplementary Table S4).

### Variation in CKD burden at the regional level

Regarding children, in 2021, the top three regions with the highest CKD incidence were Central Latin America [49.9 (43.1–58.5)], Central Asia [48.6 (40.6–57.8)], and North Africa and the Middle East [40.5 (34.8–47.9)]. On the contrary, the three regions with the lowest incidence were Western Europe [15.4 (12.2–18.9)], High-Income Asia Pacific [17.1 (13.3–21.7)], and East Asia [19.2 (15.6–23.8)]. Moreover, Western sub-Saharan Africa had the highest age-standardized mortality [2.1 (1.5–2.6)] and DALYs [189.0 (139.8–238.6)], while High-Income Asia Pacific had the lowest age-standardized mortality [0.1 (0.1–0.1)] and DALYs [13.0 (11.3–15.0)].

Among young adults in 2021, the top three regions with the highest CKD incidence were Central Latin America [70.2 (58.8–83.9)], Central Asia [67.8 (53.6–82.7)], and Eastern Europe [64.6 (47.6–83.6)]. Conversely, the three regions with the lowest incidence were Western Europe [12.0 (7.9–17.4)], Australasia [16.9 (10.9–23.9)], and Eastern sub-Saharan Africa [18.0 (14.0–22.6)]. In addition, Central sub-Saharan Africa had the highest age-standardized mortality [5.7 (4.2–7.6)] and DALYs [395.6 (303.2–509.8)], and High-Income Asia Pacific had the lowest age-standardized mortality [0.3 (0.3–0.4)] and DALYs [46.2 (37.8–56.3)].

### Correlations between SDI and burden of CKD

**Figure 2:**
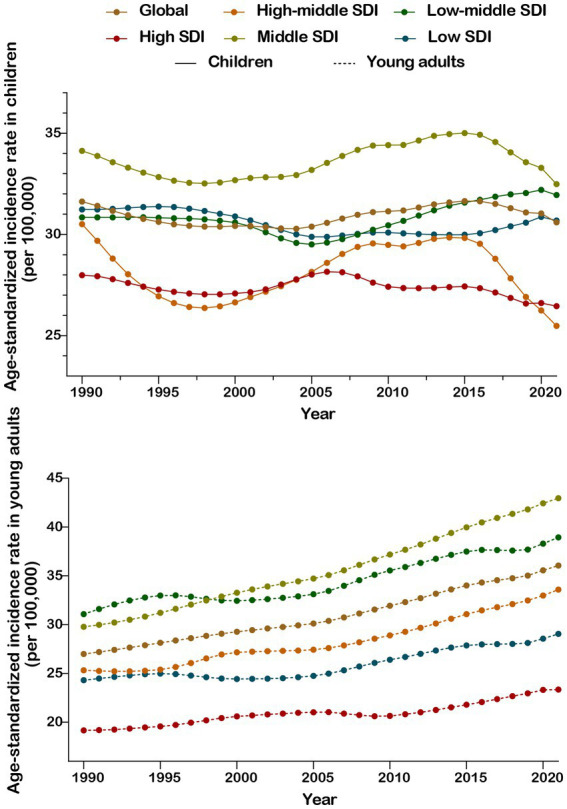
Chronic kidney disease incidence rate from 1990 to 2021 in children and young adults.

From 1990 to 2021, across regions with different SDI levels, the incidence of CKD in children fluctuated overall and trended toward stability. In contrast, the incidence of CKD in young adults showed a continuous upward trend year by year (Figure 2). Regarding mortality and DALYs, both demonstrated a downward trend among children, while in young adults, there were slight fluctuations, with a trend toward stability (Supplementary Figures S1, S2). Moreover, the CKD burden in high-middle and high SDI regions was notably lower than the global level.

**Figure 3:**
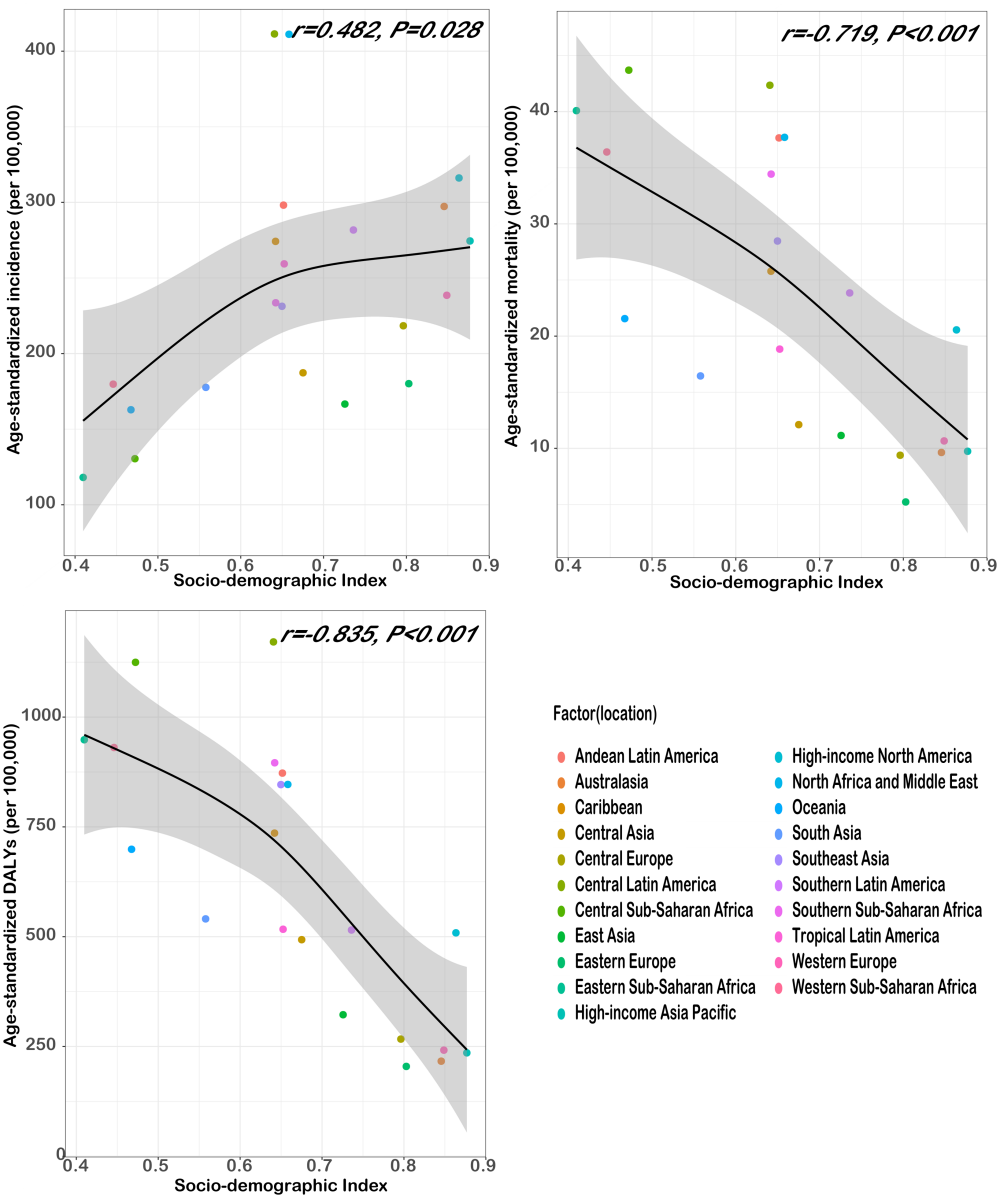
Chronic kidney disease burden in countries with different SDI levels in 2021.

A moderate positive correlation was found between age-standardized incidence and SDI (*r* = 0.482, *p* = 0.028) in 2021, indicating a relationship with an inverse U-shaped pattern. However, the age-standardized mortality (*r* = −0.719, *p* < 0.001) and DALYs (*r* = −0.835, *p* < 0.001) were strongly negatively correlated with SDI (Figure 3).

### Global burden of disease of CKD attributable to risk factors

**Figure 4:**
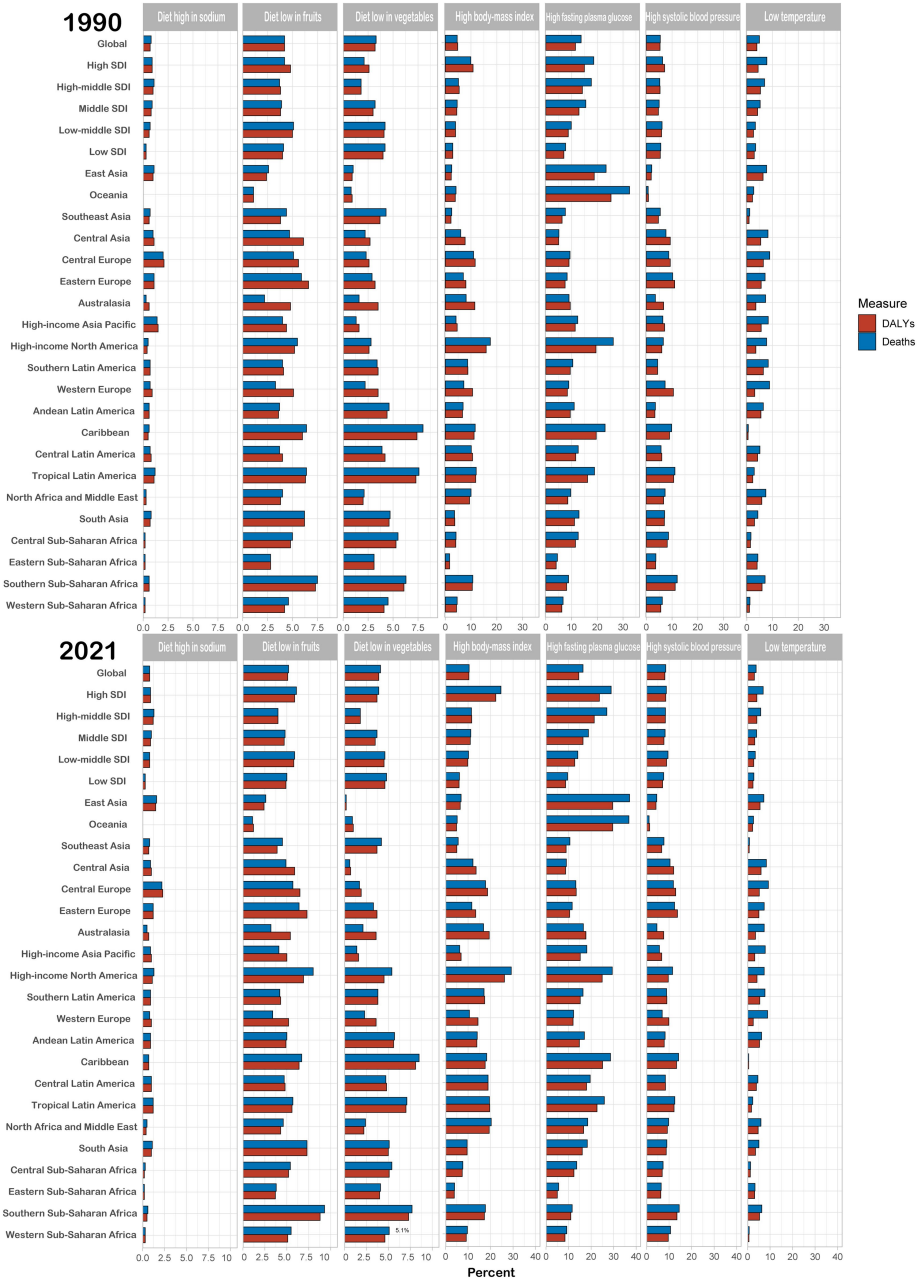
The burden of chronic kidney diseases attributable to risk factors from 1990 to 2021 in young adults.

Based on the findings of the GBD analysis (Figure 4 and Supplementary Figures S3, S4), the risk factors for death among young adults with CKD can be categorized into 14 factors across three dimensions: environmental/occupational, behavioral, and metabolic. The percentage of global total risk factors increased from 39.2% in 1990 to 53.9% in 2021. Among these factors, the metabolic risks increased from 23.7 to 35.4%, representing a 49.4% growth. Moreover, in 1990, the top seven risk factors for death were high fasting plasma glucose (HFPG), high systolic blood pressure (HSBP), low temperature (LT), high body mass index (HBMI), diet low in fruits (DLF), diet low in vegetables (DLV), and diet high in sodium (DHS), which accounted for 13.7, 5.4, 4.9, 4.6, 4.2, 3.3, and 0.8%, respectively. By 2021, the percentage of HFPG, HBMI, HSBP, DLF, and DLV had increased to 16.4, 10.5, 8.5, 5.2, and 4.1%, respectively. Meanwhile, LT decreased to 3.8%, and DHS remained constant. Geographically, in 2021, the region with the highest percentage of CKD deaths attributed to HFPG was East Asia (37.2%); for HBMI, it was High-Income North America (29.2%); for HSBP, it was Southern sub-Saharan Africa (14.5%); for DLF, it was Southern sub-Saharan Africa (9.2%); and for DLV, it was the Caribbean (8.5%). When examining attributable risk factors according to the SDI level, it was discovered that the higher the SDI level, the higher the risk percentages of HFPG and HBMI. Additionally, the death risk percentage attributed to a diet high in processed meat and red meat in High SDI regions was significantly higher than in other regions.

Regarding the death risk factors of CKD in children, this study identified only two risk factors, namely high and low temperature, as shown in Supplementary Figure S5.

### Future forecasts of the global burden of disease of CKD

**Figure 5:**
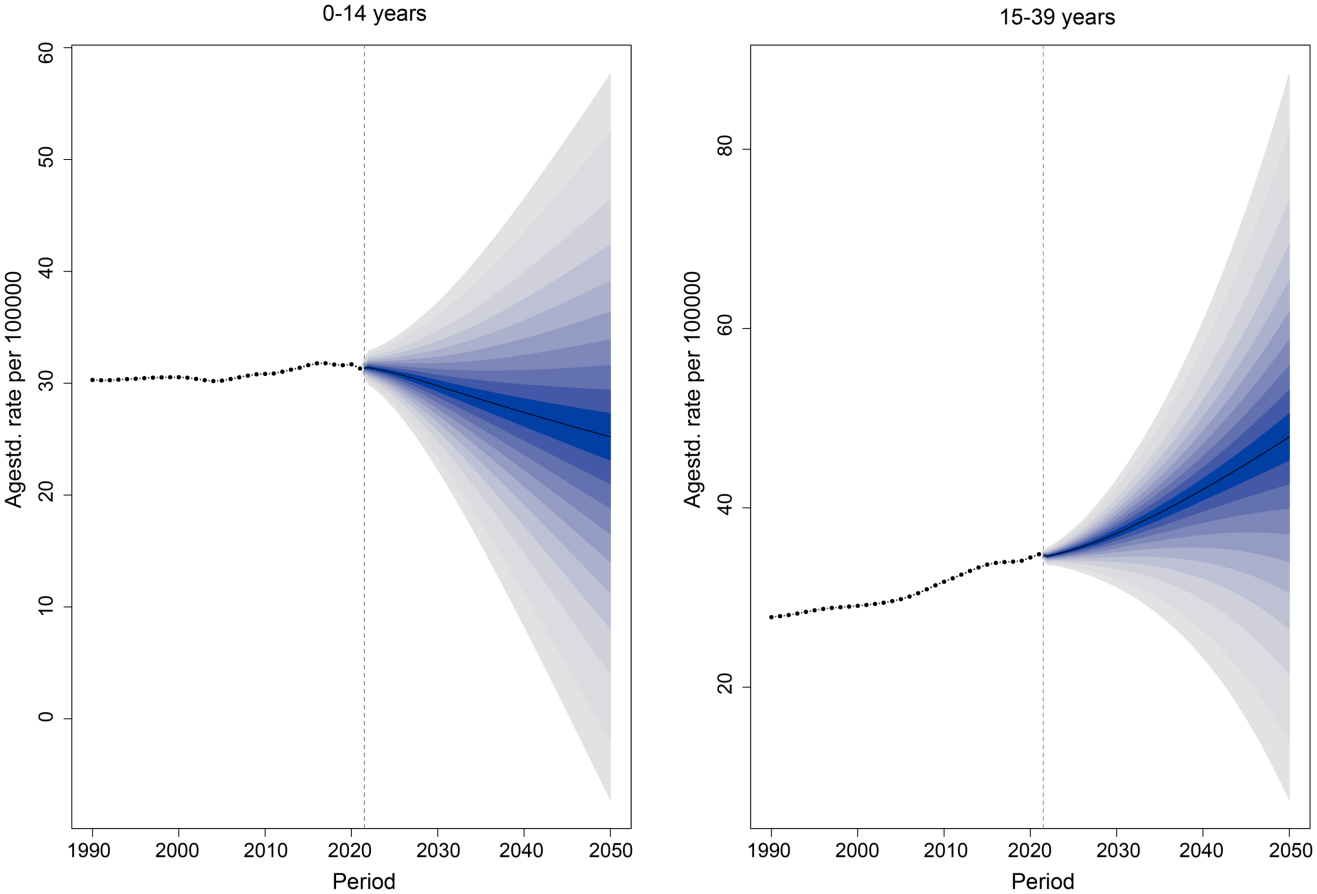
Future forecast of chronic kidney disease-related incidence rate. The dotted line indicates 2021, the actual incidence on the left side of the dotted line, and the predicted incidence on the right side of the dotted line. The solid black line in dark blue is the predicted trend of incidence.

In this study, a comprehensive projection of the global incidence of CKD in both children and young adults from 2022 to 2050 was meticulously carried out. As shown in Figure 5, the future trends of CKD incidence are distinct for the two age groups, showing a downward trajectory in children and an upward one in young adults. Specifically, it is estimated that, by 2050, the number of incident cases of CKD in children will reach 552,469.7, corresponding to an incidence rate of 25.20 per 100,000 population, representing a 17.65% reduction compared to 2021. When analyzed by age subgroups, the incidence rates are 62.19 per 100,000 for children under 5 years, 12.48 per 100,000 for those aged 5–9 years, and 17.80 per 100,000 for those aged 10–14 years, corresponding to an increase of 1.66%, a decrease of 4.32%, and an increase of 0.19%, respectively, compared with 2021, as shown in Supplementary Figure S6. For young adults, the incidence rate of CKD is projected to increase from 36.06 per 100,000 in 2021 to 47.92 per 100,000 in 2050, with incident cases reaching 1,768,601. When segmented by age, the incidence rates for different age groups are 37.10 per 100,000 (15–19 years), 36.15 per 100,000 (20–24 years), 40.12 per 100,000 (25–29 years), 59.23 per 100,000 (30–34 years), and 99.58 per 100,000 (35–39 years), corresponding to increases of 68.69, 67.34, 63.05, 50.31, and 37.76%, respectively, compared with 2021.

## Discussion

In general, the CKD burden in children shows a gradual decline, whereas in young adults, it is increasing rapidly. Geographically, Western Europe and High-Income Asia-Pacific regions have a relatively low CKD burden, in contrast to the heavier burden observed in Latin America, Central Asia, North Africa, and the Middle East. Based on SDI, further analysis reveals that, compared to low SDI regions, high SDI regions have lower mortality and DALYs. For young adults, the primary risk factors for CKD-related deaths are metabolic factors, specifically HFPG, HSBP, and HBMI. Projections indicate that, from 2022 to 2050, the incidence of CKD in children is expected to decline markedly, whereas the upward trend among young adults is expected to persist.

CKD is a chronic disease with severe long-term consequences, and it is more common among young adults. Although young adults and children with CKD share some similarities in terms of etiology and symptoms, there are unique features in children not evident in adults. For example, the main causes of CKD in children are congenital abnormalities of the kidney and urinary tract (CAKUT), steroid-resistant nephrotic syndrome (SRNS), and glomerulonephritis (17–19). Notably, the mortality rate among children with end-stage renal disease who are undergoing dialysis is 30 to 150 times higher than that of the general pediatric population, and life expectancy for a child on dialysis is approximately 50 years less than that of a healthy child (20). Additionally, the most important goal for these children is to have a normal childhood. In young adults, CKD stems from multiple etiologies, such as diabetes, glomerulonephritis, cystic kidney disease, hypertension, obesity, and air pollution, although the causation is not yet fully understood (21–24). Common mental disorders such as depression, anxiety, and cognitive impairment frequently affect CKD patients (25). In particular, the prevalence of depression is two to three times higher in patients with renal failure compared with other chronically ill populations, such as those with diabetes mellitus, ischemic heart disease, cerebrovascular disease, and peptic ulcer disease (26). In daily lives, CKD patients face substantial limitations: they miss social opportunities, lack social confidence and skills, and experience profound uncertainty about their future (27).

Our analysis of the GBD 2021 study reveals that the burden of CKD in children is reflected primarily in declining mortality and DALYs. Notably, we observed significant downward trends during two distinct periods (1990–1995 and 2017–2021), spanning a total of 11 years. Consistent with previous findings, Brazil has demonstrated a sustained decline in childhood CKD mortality since 1990, particularly among children aged 5–15 years, a progress attributable to improvements in the healthcare system and socioeconomic development (28). Geospatial analysis identified Central Latin America, Central Asia, North Africa, and the Middle East as regions with the highest child CKD burdens, whereas Western Europe, the High-Income Asia Pacific, and East Asia showed the lowest incidence. This geographic disparity underscores the crucial link between socioeconomic status and the burden of CKD. High-SDI regions benefit from robust healthcare infrastructure coupled with enhanced levels of health literacy and education. Conversely, low-SDI regions face a concerning pattern of therapeutic discontinuation due to financial constraints, often leading to rapid health deterioration. This phenomenon is called “near-suicide.” (29) Regarding mortality risk factors, while our study lacked sufficient data for comprehensive analysis, existing evidence identifies cardiovascular diseases as the predominant cause of death in pediatric CKD patients (30). Key modifiable risk factors include hypertension, dyslipidemia, obesity, hyperglycemia, anemia, hyperparathyroidism, and hypoalbuminemia (31). Notably, despite persistent disparities in regional burden distribution, the global trend indicates a progressive reduction in the CKD burden among children. This optimistic projection holds particular significance for economically disadvantaged regions currently experiencing a disproportionate disease impact.

A concentrated manifestation of CKD burden among young adults is the dramatically escalating incidence rates. From 1990 to 2021, we observed a persistent upward trajectory, with the most pronounced surge occurring between 2019 and 2021. The trend will intensify in the future. This epidemiological pattern primarily stems from the growing prevalence of hypertension, obesity, and type 2 diabetes mellitus in younger populations. Geospatial analysis identifies Central Latin America, Central Asia, and Eastern Europe as regions bearing the highest CKD burdens, whereas Western Europe, Australasia, and Southern Latin America demonstrate the lowest incidence. This geographical stratification mirrors socioeconomic disparities, with previous studies establishing a direct correlation between gross national income per capita and access to renal replacement therapy (RRT) (32, 33). Economic prosperity facilitates higher dialysis and renal transplantation rates. At the same time, developing nations face dual challenges: prohibitive RRT costs compounded by critical shortages of nephrology specialists, nursing staff, and dialysis facilities (34, 35). In addition, RRT may also be a cultural issue reflecting the fundamental values of a society. For example, the concept of life preservation, which becomes embedded in cultural frameworks and subsequently informs health policy (34, 35). Our risk factor analysis identifies HFPG, HBMI, HSBP, DLF, and DLV as the primary determinants of mortality in young adults with CKD. Intriguingly, regions with higher SDI levels demonstrate greater attributable risk proportions for HFPG, HBMI, and diets high in processed and red meat—a paradoxical phenomenon highlighting both material affluence and nutritional risks in developed countries. The glomerulus, the filtering unit of the kidney, is particularly susceptible to barotrauma. Diets rich in animal protein not only impair glomerular self-protective mechanisms but also promote net endogenous acid production (36, 37). Conversely, fruits and vegetables offer multi-modal protection: they neutralize metabolic acidosis, improve gut dysbiosis, and reduce the number of pathobionts and protein-fermenting species, thereby decreasing the production of the most harmful uremic toxins. The high fiber content of these diets enhances intestinal motility and short-chain fatty acid production (36, 37). Therefore, dietary modification should be regarded as a crucial intervention strategy to prevent CKD progression.

To our knowledge, this study, grounded in the GBD framework, represents a pioneering effort to comprehensively analyze global CKD incidence, mortality, DALYs, risk factors, and future trends among children and young adults. At the same time, it contributes valuable evidence to global CKD research and informs prevention strategies for the occurrence and development of CKD. However, this study also has several limitations. First, the accuracy of our findings depends fundamentally on the quality of the GBD 2021 study. Many countries do not have high-quality population-based studies on the occurrence of CKD. Second, the GBD 2021 provides only certain environmental risks, behavioral risks, and metabolic risks, and the risk factors associated with death in children are insufficient. Third, we described the total incidence of all CKD stages in children and young adults but could not provide detailed information on CKD by each stage. Fourth, GBD data are continuously updated, so this study represents the CKD burden only for the stated period, highlighting the need for future updates. Addressing these limitations in subsequent GBD studies will enhance the estimates of the CKD burden in children and young adults and provide a strong basis for policy and clinical decision-making.

In conclusion, we comprehensively assessed the burden, risk factors, and future trends of CKD in children and young adults from global, regional, and SDI perspectives. Globally, CKD is still a major health problem and is expected to continue increasing over the next two decades, especially among young adults. High SDI regions should remain mindful of the importance of a balanced diet in slowing CKD progression, while the broader society should provide greater attention, support, and resources to low SDI regions.

## Data Availability

The original contributions presented in the study are included in the article/Supplementary material, further inquiries can be directed to the corresponding authors.

## Footnotes

1
https://ghdx.healthdata.org/gbd-2021

2
https://ghdx.healthdata.org/record/ihme-data/global-burden-disease-study-2021-gbd-2021-demographics-1950-2021

3
https://ghdx.healthdata.org/record/ihme-data/global-population-forecasts-2017-2100
