# Supplementary material for: Global trends and risk factors of chronic kidney disease in children and young adults from 1990 to 2021: a systematic analysis of the global burden of disease study 2021
Source: Front Public Health. 2025 Dec 18;13:1696021. doi: 10.3389/fpubh.2025.1696021 (PMC12756421; doi:10.3389/fpubh.2025.1696021)
Supplement: Supplementary file 1 [file Supplementary_file_1.docx]

**Supplementary material**

**Global trends and risk factors of chronic kidney disease in children and young adults from 1990 to 2021: a systematic analysis of Global Burden of Disease Study 2021**

**Supplemental table 1 Age-standardized mortality rate for chronic kidney disease in children and young adults from 1990 to 2021.**

| **Characteristics** | **Age-standardized death rate per 100,000 population in 1990 (95%UI)** | | **Age-standardized death rate per 100,000 population in 2021 (95%UI)** | | **Percentage change from 1990 to 2021, % (95%UI)** | |
| --- | --- | --- | --- | --- | --- | --- |
|  | **0-14 years** | **15-39 years** | **0-14 years** | **15-39 years** | **0-14 years** | **15-39 years** |
| **Global** | 1.5 (1.0 - 1.7) | 2.6 (2.3 - 2.9) | 0.8 (0.6 - 0.9) | 2.8 (2.5 - 3.1) | -45.1 (-54.3 - -24.0)^*^ | 8.0 (-6.0 - 21.3) |
| Male | 1.6 (1.0 - 2.0) | 2.8 (2.4 - 3.2) | 0.9 (0.7 - 1.1) | 3.2 (2.8 - 3.6) | -45.5 (-56.5 - -12.2)^*^ | 14.9 (-2.7 - 31.1) |
| Female | 1.3 (1.0 - 1.5) | 2.3 (2.1 - 2.6) | 0.7 (0.6 - 0.8) | 2.3 (2.1 - 2.6) | -44.5 (-55.1 - -26.1)^*^ | -0.8 (-18.2 - 15.5) |
| **SDI groups** |  |  |  |  |  |  |
| High SDI | 0.6 (0.6 - 0.6) | 0.8 (0.8 - 0.9) | 0.3 (0.3 - 0.3) | 1.1 (0.9 - 1.3) | -48.8 (-53.5 - -43.6)^*^ | 32.2 (7.1 - 54.7)^*^ |
| High-middle SDI | 0.8 (0.7 - 0.9) | 1.9 (1.8 - 2.2) | 0.3 (0.2 - 0.3) | 1.4 (1.2 - 1.6) | -65.9 (-70.8 - -60.4)^*^ | -28.9 (-42.0 - -18.2)^*^ |
| Middle SDI | 1.3 (1.0 - 1.4) | 3.2 (2.8 - 3.5) | 0.6 (0.5 - 0.6) | 3.1 (2.8 - 3.4) | -56.2 (-62.4 - -46.9)^*^ | -1.1 (-16.3 - 11.9) |
| Low-middle SDI | 1.9 (1.1 - 2.4) | 2.9 (2.5 - 3.3) | 0.9 (0.7 - 1.1) | 3.2 (2.8 - 3.6) | -50.9 (-62.4 - -17.8)^*^ | 11.2 (-8.7 - 31.6) |
| Low SDI | 2.5 (1.8 - 3.0) | 4.2 (3.6 - 4.9) | 1.4 (1.1 - 1.7) | 4.0 (3.3 - 4.8) | -45.0 (-55.4 - -20.1)^*^ | -5.2 (-18.2 - 9.8) |
| **Geographic regions** |  |  |  |  |  |  |
| East Asia | 1.0 (0.8 - 1.2) | 2.6 (2.3 - 3.0) | 0.2 (0.2 - 0.3) | 1.6 (1.3 - 2.0) | -80.7 (-84.2 - -73.7)^*^ | -37.7 (-53.3 - -20.8)^*^ |
| Oceania | 1.1 (0.5 - 1.5) | 3.2 (1.8 - 4.5) | 1.3 (0.8 - 1.8) | 4.1 (3.1 - 5.1) | 23.6 (-18.4 - 95.5) | 28.7 (-10.9 - 104.7) |
| Southeast Asia | 1.6 (1.0 - 1.9) | 5.1 (4.0 - 5.8) | 0.9 (0.7 - 1.1) | 5.4 (4.2 - 6.3) | -43.0 (-54.3 - -16.0)^*^ | 7.0 (-10.4 - 27.7) |
| Central Asia | 0.4 (0.3 - 0.4) | 2.2 (2.0 - 2.4) | 0.6 (0.5 - 0.7) | 3.5 (3.0 - 3.9) | 45.4 (16.2 - 88.2)^*^ | 59.9 (38.2 - 82.8)^*^ |
| Central Europe | 1.0 (0.9 - 1.0) | 1.6 (1.6 - 1.7) | 0.2 (0.2 - 0.3) | 0.8 (0.7 - 0.9) | -75.1 (-79.1 - -69.6)^*^ | -52.7 (-58.7 - -44.7)^*^ |
| Eastern Europe | 0.4 (0.4 - 0.4) | 1.8 (1.8 - 1.8) | 0.2 (0.2 - 0.2) | 1.0 (0.9 - 1.1) | -53.8 (-58.0 - -47.8)^*^ | -44.2 (-49.7 - -38.3)^*^ |
| Australasia | 0.7 (0.7 - 0.8) | 0.3 (0.3 - 0.3) | 0.4 (0.3 - 0.4) | 0.4 (0.3 - 0.4) | -51.9 (-61.1 - -41.1)^*^ | 15.7 (6.9 - 22.8)^*^ |
| High-income Asia Pacific | 0.3 (0.3 - 0.4) | 0.9 (0.8 - 1.0) | 0.1 (0.1 - 0.1) | 0.3 (0.3 - 0.4) | -68.6 (-72.5 - -62.7)^*^ | -63.2 (-66.6 - -50.9)^*^ |
| High-income North America | 0.8 (0.7 - 0.8) | 0.6 (0.6 - 0.7) | 0.4 (0.4 - 0.5) | 1.2 (1.1 - 1.3) | -45.0 (-51.1 - -38.3)^*^ | 85.6 (65.2 - 97.9)^*^ |
| Southern Latin America | 0.9 (0.8 - 0.9) | 2.0 (1.9 - 2.1) | 0.4 (0.3 - 0.5) | 1.4 (1.3 - 1.5) | -55.3 (-62.5 - -46.9)^*^ | -32.0 (-37.5 - -26.0)^*^ |
| Western Europe | 0.4 (0.4 - 0.4) | 0.3 (0.3 - 0.4) | 0.3 (0.2 - 0.3) | 0.3 (0.3 - 0.3) | -39.3 (-47.1 - -31.1)^*^ | -23.4 (-28.1 - -15.7)^*^ |
| Andean Latin America | 2.4 (2.1 - 2.8) | 3.5 (3.1 - 4.0) | 0.9 (0.8 - 1.2) | 3.0 (2.5 - 3.7) | -61.1 (-69.8 - -49.5)^*^ | -13.4 (-31.6 - 7.6) |
| Caribbean | 1.9 (1.4 - 2.2) | 3.0 (2.7 - 3.5) | 1.5 (1.1 - 2.1) | 4.3 (3.4 - 5.9) | -20.4 (-40.4 - 8.6) | 44.7 (18.9 - 77.2)^*^ |
| Central Latin America | 1.7 (1.6 - 1.8) | 3.4 (3.4 - 3.5) | 0.8 (0.6 - 0.9) | 5.4 (4.8 - 6.2) | -55.1 (-63.5 - -44.3)^*^ | 58.5 (41.6 - 77.7)^*^ |
| Tropical Latin America | 1.2 (1.1 - 1.4) | 2.6 (2.5 - 2.7) | 0.5 (0.4 - 0.6) | 1.7 (1.6 - 1.8) | -58.3 (-67.8 - -46.3)^*^ | -34.6 (-39.3 - -30.3)^*^ |
| North Africa and Middle East | 1.8 (1.3 - 2.3) | 2.5 (2.1 - 3.3) | 0.9 (0.7 - 1.1) | 2.9 (2.4 - 3.4) | -50.1 (-62.4 - -28.0)^*^ | 15.0 (-20.8 - 49.1) |
| South Asia | 1.5 (0.7 - 1.9) | 2.0 (1.8 - 2.4) | 0.6 (0.4 - 0.7) | 2.1 (1.7 - 2.6) | -60.3 (-70.7 - -20.1)^*^ | 5.7 (-24.3 - 30.0) |
| Central Sub-Saharan Africa | 2.9 (2.1 - 3.8) | 5.6 (4.3 - 7.0) | 1.2 (0.8 - 2.5) | 5.7 (4.2 - 7.6) | -59.7 (-71.0 - -32.6)^*^ | 0.9 (-26.4 - 40.4) |
| Eastern Sub-Saharan Africa | 2.5 (1.7 - 3.0) | 5.6 (4.7 - 6.4) | 1.2 (0.9 - 1.5) | 4.9 (4.1 - 5.9) | -52.3 (-63.7 - -19.4)^*^ | -11.7 (-25.2 - 6.5) |
| Southern Sub-Saharan Africa | 0.8 (0.7 - 1.0) | 3.7 (3.3 - 4.4) | 0.9 (0.7 - 1.1) | 4.4 (3.8 - 5.2) | 3.3 (-17.3 - 31.2) | 17.5 (-2.0 - 39.9) |
| Western Sub-Saharan Africa | 3.6 (2.5 - 4.4) | 4.6 (3.6 - 5.8) | 2.1 (1.5 - 2.6) | 4.7 (3.5 - 6.0) | -42.6 (-54.8 - -23.3)^*^ | 1.7 (-16.1 - 21.1) |

^*^:percentage change is significant.

**Supplemental table 2 Age-standardized DALY rate for chronic kidney disease in children and young adults from 1990 to 2021.**

| **Characteristics** | **Age-standardized DALY rate per 100,000 population in 1990 (95%UI)** | | **Age-standardized DALY rate per 100,000 population in 2021 (95%UI)** | | **Percentage change from 1990 to 2021, % (95%UI)** | |
| --- | --- | --- | --- | --- | --- | --- |
|  | **0-14 years** | **15-39 years** | **0-14 years** | **15-39 years** | **0-14 years** | **15-39 years** |
| **Global** | 132.6 (96.1 - 151.1) | 198.3 (176.8 - 223.9) | 75.3 (60.8 - 87.2) | 211.1 (188.7 - 236.7) | -43.2 (-52.6 - -22.7)^*^ | 6.5 (-4.5 - 16.9) |
| Male | 149.4 (94.6 - 177.7) | 210.8 (181.3 - 243.9) | 84.0 (66.1 - 98.3) | 237.5 (208.0 - 269.1) | -43.8 (-54.4 - -12.3)^*^ | 12.6 (-1.8 - 25.8) |
| Female | 114.8 (93.1 - 135.9) | 185.4 (163.3 - 212.0) | 65.9 (56.8 - 76.6) | 183.9 (162.6 - 208.6) | -42.6 (-52.7 - -24.6)^*^ | -0.8 (-15.0 - 11.3) |
| **SDI groups** |  |  |  |  |  |  |
| High SDI | 57.6 (54.9 - 61.3) | 86.1 (72.8 - 103.4) | 32.3 (29.1 - 35.7) | 105.3 (86.4 - 125.0) | -44.0 (-48.7 - -39.3)^*^ | 22.3 (7.6 - 35.7)^*^ |
| High-middle SDI | 72.5 (63.7 -81.3) | 152.5 (133.7 - 176.9) | 27.3 (24.1 - 31.1) | 115.7 (100.6 - 135.5) | -62.3 (-67.2 - -57.0)^*^ | -24.1 (-34.8 - -16.2)^*^ |
| Middle SDI | 114.7 (91.6 - 127.5) | 238.1 (209.1 - 271.8) | 52.7 (45.2 - 60.5) | 234.7 (206.4 - 260.6) | -54.0 (-60.2 - -44.6)^*^ | -1.4 (-13.1 - 8.4) |
| Low-middle SDI | 174.1 (99.9 - 214.1) | 221.4 (194.5 - 257.2) | 88.3 (70.6 - 103.5) | 241.1 (213.7 - 272.7) | -49.3 (-60.4 - -16.2)^*^ | 8.9 (-7.3 - 24.1) |
| Low SDI | 224.8 (164.1 - 266.2) | 302.5 (264.4 - 348.9) | 126.8 (98.7 - 154.6) | 285.2 (241.4 - 341.5) | -43.6 (-54.1 - -20.0)^*^ | -5.7 (-17.3 - 7.5) |
| **Geographic regions** |  |  |  |  |  |  |
| East Asia | 93.0 (73.9 - 107.1) | 193.9 (165.4 - 224.8) | 19.1 (16.1 - 23.2) | 125.0 (102.6 - 152.3) | -79.5 (-83.0 - -72.6)^*^ | -35.5 (-48.8 - -21.8)^*^ |
| Oceania | 98.4 (50.0 - 137.2) | 238.3 (152.9 - 320.1) | 120.0 (73.9 - 162.5) | 296.5 (229.8 - 370.8) | 22.0 (-17.6 - 84.0) | 24.4 (-8.6 - 78.4) |
| Southeast Asia | 144.3 (94.1 - 171.4) | 363.5 (293.3 - 413.9) | 83.5 (68.0 - 98.2) | 380.0 (309.5 - 441.2) | -42.2 (-52.8 - -16.9)^*^ | 4.6 (-10.3 - 21.8) |
| Central Asia | 47.0 (38.4 - 57.0) | 205.1 (177.1 - 239.2) | 61.6 (50.7 - 74.5) | 288.1 (250.7 - 337.6) | 31.2 (11.3 - 62.7)^*^ | 40.5 (27.0 - 56.3)^*^ |
| Central Europe | 99.7 (91.1 - 110.3) | 131.9 (118.8 - 149.0) | 31.8 (26.1 - 39.9) | 78.6 (65.2 - 97.0) | -68.1 (-73.0 - -62.5)^*^ | -40.4 (-46.3 - -33.1)^*^ |
| Eastern Europe | 41.0 (37.9 - 46.3) | 136.9 (126.0 - 151.6) | 21.8 (18.7 - 26.4) | 85.6 (73.6 - 100.4) | -46.8 (-51.6 - -40.4)^*^ | -37.5 (-43.0 - -32.1)^*^ |
| Australasia | 68.5 (61.2 - 76.6) | 39.7 (32.0 - 51.0) | 35.1 (29.2 - 42.1) | 46.2 (36.5 - 59.2) | -48.8 (-58.1 - -37.9)^*^ | 16.4 (7.3 - 27.8)^*^ |
| High-income Asia Pacific | 32.2 (29.6 - 35.5) | 78.6 (69.3 - 90.1) | 13.0 (11.3 - 15.0) | 46.2 (37.8 - 56.3) | -59.5 (-64.7 - -52.7)^*^ | -41.2 (-47.9 - -31.1)^*^ |
| High-income North America | 73.6 (69.8 - 79.2) | 82.8 (67.0 - 102.5) | 43.5 (38.9 - 48.5) | 121.7 (102.4 - 144.6) | -40.9 (-46.9 - -34.2)^*^ | 47.1 (34.7 - 59.8)^*^ |
| Southern Latin America | 81.3 (76.9 - 85.4) | 155.0 (142.1 - 170.7) | 39.0 (33.3 - 45.7) | 116.6 (103.5 - 132.4) | -52.0 (-58.7 - -43.7)^*^ | -24.8 (-30.6 - -19.1)^*^ |
| Western Europe | 42.4 (40.6 - 44.8) | 59.0 (44.9 - 75.6) | 28.0 (24.8 - 31.8) | 56.5 (41.4 - 75.3) | -33.9 (-41.5 - -26.2)^*^ | -4.2 (-10.9 - 2.6) |
| Andean Latin America | 218.7 (190.6 - 247.2) | 253.0 (223.7 - 285.2) | 88.1 (72.2 - 106.1) | 216.4 (179.7 - 259.3) | -59.7 (-68.0 - -48.9)^*^ | -14.5 (-30.0 - 3.1) |
| Caribbean | 167.9 (129.5 - 201.0) | 216.5 (194.7 - 250.5) | 135.4 (99.2 - 184.7) | 297.6 (243.3 - 394.0) | -19.4 (-38.3 - 7.9) | 37.5 (16.5 - 64.9)^*^ |
| Central Latin America | 154.9 (145.2 - 166.8) | 256.9 (238.8 - 280.9) | 72.3 (59.8 - 86.5) | 377.8 (335.3 - 428.7) | -53.3 (-61.6 - -43.0)^*^ | 47.1 (33.1 - 62.7)^*^ |
| Tropical Latin America | 112.9 (101.2 - 125.4) | 197.4 (182.2 - 216.9) | 49.6 (40.2 - 59.3) | 138.6 (123.6 - 157.3) | -56.1 (-65.4 - -44.6)^*^ | -29.8 (-34.9 - -25.2)^*^ |
| North Africa and Middle East | 164.0 (116.5 - 204.2) | 196.0 (164.9 - 247.4) | 84.7 (71.1 - 100.0) | 214.4 (184.8 - 251.2) | -48.4 (-60.1 - -26.7)^*^ | 9.4 (-19.1 - 35.1) |
| South Asia | 133.1 (65.5 - 169.8) | 172.9 (148.0 - 206.1) | 55.9 (44.8 - 65.4) | 182.4 (153.2 - 220.7) | -58.0 (-68.4 - -19.3)^*^ | 5.5 (-15.8 - 22.7) |
| Central Sub-Saharan Africa | 264.2 (194.5 - 339.2) | 398.2 (309.4 - 489.4) | 110.3 (76.3 - 225.6) | 395.6 (303.2 - 509.8) | -58.3 (-69.2 - -32.2)^*^ | -0.7 (-25.4 - 35.4) |
| Eastern Sub-Saharan Africa | 220.9 (154.2 - 262.6) | 379.2 (329.4 - 434.5) | 108.3 (83.2 - 141.1) | 332.8 (281.9 - 404.3) | -51.0 (-62.0 - -19.5)^*^ | -12.2 (-24.9 - 5.0) |
| Southern Sub-Saharan Africa | 80.3 (65.4 - 93.8) | 261.6 (229.4 - 309.5) | 82.4 (69.7 - 98.9) | 300.2 (260.6 - 351.7) | 2.5 (-16.1 - 27.1) | 14.8 (-1.8 - 34.1) |
| Western Sub-Saharan Africa | 321.5 (227.7 - 389.9) | 326.4 (263.6 - 406.6) | 189.0 (139.8 - 238.6) | 330.6 (252.9 - 420.1) | -41.2 (-53.2 - -22.8)^*^ | 1.3 (-14.4 - 17.9) |

^*^:percentage change is significant.

**Supplemental table 3 Trends of global CKD burden in children during 1990-2021.**

| **Measure** | **Period** | **APC/AAPC** | **Lower CI** | **Upper CI** | ***t*** | ***P*** |
| --- | --- | --- | --- | --- | --- | --- |
| Incidence | 1990-1995 | -0.687* | -0.808 | -0.566 | -11.864 | < 0.000001 |
|  | 1995-2004 | -0.104* | -0.161 | -0.047 | -3.854 | 0.001163 |
|  | 2004-2008 | 0.574* | 0.332 | 0.816 | 4.998 | 0.000093 |
|  | 2008-2016 | 0.303* | 0.239 | 0.367 | 9.918 | < 0.000001 |
|  | 2016-2021 | -0.653* | -0.765 | -0.541 | -12.202 | < 0.000001 |
|  | 1990-2021 | -0.095*^#^ | -0.139 | -0.051 | -4.240 | 0.000022 |
| Prevalence | 1990-1993 | 0.149* | 0.040 | 0.258 | 2.877 | 0.010022 |
|  | 1993-2000 | 0.679* | 0.641 | 0.717 | 37.738 | < 0.000001 |
|  | 2000-2010 | -0.829* | -0.851 | -0.808 | -81.695 | < 0.000001 |
|  | 2010-2018 | -0.217* | -0.249 | -0.184 | -14.022 | < 0.000001 |
|  | 2018-2021 | 0.547* | 0.422 | 0.673 | 9.213 | < 0.000001 |
|  | 1990-2021 | -0.104*^#^ | -0.124 | -0.085 | -10.390 | < 0.000001 |
| Mortality | 1990-1998 | -2.196* | -2.365 | -2.027 | -27.985 | < 0.000001 |
|  | 1998-2003 | -1.811* | -2.238 | -1.382 | -9.134 | 0.000001 |
|  | 2003-2006 | -0.606 | -1.869 | 0.673 | -1.036 | 0.320679 |
|  | 2006-2011 | -1.360* | -1.711 | -1.008 | -8.367 | 0.000002 |
|  | 2011-2014 | -0.577 | -1.708 | 0.568 | -1.100 | 0.292771 |
|  | 2014-2017 | -1.990* | -3.122 | -0.846 | -3.773 | 0.002658 |
|  | 2017-2021 | -4.138* | -4.550 | -3.726 | -21.425 | < 0.000001 |
|  | 1990-2021 | -1.925*^#^ | -2.129 | -1.720 | -18.301 | < 0.000001 |
| DALYs | 1990-1998 | -2.117* | -2.266 | -1.969 | -30.754 | < 0.000001 |
|  | 1998-2003 | -1.699* | -2.074 | -1.322 | -9.763 | < 0.000001 |
|  | 2003-2006 | -0.635 | -1.770 | 0.512 | -1.209 | 0.249847 |
|  | 2006-2011 | -1.335* | -1.650 | -1.020 | -9.174 | 0.000001 |
|  | 2011-2014 | -0.487 | -1.486 | 0.521 | -1.056 | 0.311982 |
|  | 2014-2017 | -1.871* | -2.892 | -0.839 | -3.935 | 0.001979 |
|  | 2017-2021 | -3.799* | -4.157 | -3.439 | -22.590 | < 0.000001 |
|  | 1990-2021 | -1.820*^#^ | -2.002 | -1.638 | -19.431 | < 0.000001 |

^#^, refer to the AAPC; *, *P*<0.05; CI, confidence interval; DALYs, disability-adjusted life years; APC, annual percentage change; AAPC, average annual percentage change

**Supplemental table 4 Trends of global CKD burden in young adults during 1990-2021.**

| **Measure** | **Period** | **APC/AAPC** | **Lower CI** | **Upper CI** | ***t*** | ***P*** |
| --- | --- | --- | --- | --- | --- | --- |
| Incidence | 1990-1999 | 0.837* | 0.823 | 0.850 | 126.642 | < 0.000001 |
|  | 1999-2006 | 0.606* | 0.582 | 0.631 | 51.540 | < 0.000001 |
|  | 2006-2015 | 1.285* | 1.270 | 1.300 | 179.959 | < 0.000001 |
|  | 2015-2019 | 0.732* | 0.663 | 0.801 | 22.456 | < 0.000001 |
|  | 2019-2021 | 1.458* | 1.320 | 1.596 | 22.382 | < 0.000001 |
|  | 1990-2021 | 0.941*^#^ | 0.927 | 0.955 | 132.606 | < 0.000001 |
| Prevalence | 1990-2000 | 0.475* | 0.456 | 0.495 | 52.163 | < 0.000001 |
|  | 2000-2005 | -0.241* | -0.318 | -0.164 | -6.555 | 0.000004 |
|  | 2005-2010 | 0.361* | 0.284 | 0.438 | 9.853 | < 0.000001 |
|  | 2010-2015 | 0.761* | 0.683 | 0.839 | 20.562 | < 0.000001 |
|  | 2015-2021 | 0.287* | 0.244 | 0.329 | 14.161 | < 0.000001 |
|  | 1990-2021 | 0.350*^#^ | 0.328 | 0.373 | 30.760 | < 0.000001 |
| Mortality | 1990-1994 | 0.764* | 0.274 | 1.256 | 3.401 | 0.005259 |
|  | 1994-1997 | -0.013 | -1.433 | 1.427 | -0.020 | 0.984313 |
|  | 1997-2000 | 1.681* | 0.270 | 3.112 | 2.599 | 0.023264 |
|  | 2000-2004 | -0.205 | -0.803 | 0.396 | -0.745 | 0.470797 |
|  | 2004-2007 | -1.514* | -2.472 | -0.546 | -3.399 | 0.005280 |
|  | 2007-2012 | -0.291 | -0.599 | 0.019 | -2.045 | 0.063429 |
|  | 2012-2021 | 0.755* | 0.640 | 0.871 | 14.325 | < 0.000001 |
|  | 1990-2021 | 0.256*^#^ | 0.035 | 0.477 | 2.274 | 0.022962 |
| DALYs | 1990-1994 | 0.529* | 0.176 | 0.883 | 3.268 | 0.006731 |
|  | 1994-1997 | 0.049 | -1.054 | 1.164 | 0.096 | 0.924925 |
|  | 1997-2000 | 1.283* | 0.232 | 2.344 | 2.663 | 0.020664 |
|  | 2000-2004 | -0.086 | -0.602 | 0.432 | -0.364 | 0.722223 |
|  | 2004-2007 | -1.223* | -2.189 | -0.248 | -2.730 | 0.018266 |
|  | 2007-2012 | -0.124 | -0.432 | 0.184 | -0.878 | 0.397414 |
|  | 2012-2021 | 0.485* | 0.389 | 0.582 | 10.969 | < 0.000001 |
|  | 1990-2021 | 0.187*^#^ | 0.005 | 0.368 | 2.018 | 0.043565 |

^#^, refer to the AAPC; *, *P*<0.05 DALYs, disability-adjusted life years; APC, annual percentage change; AAPC, average annual percentage change


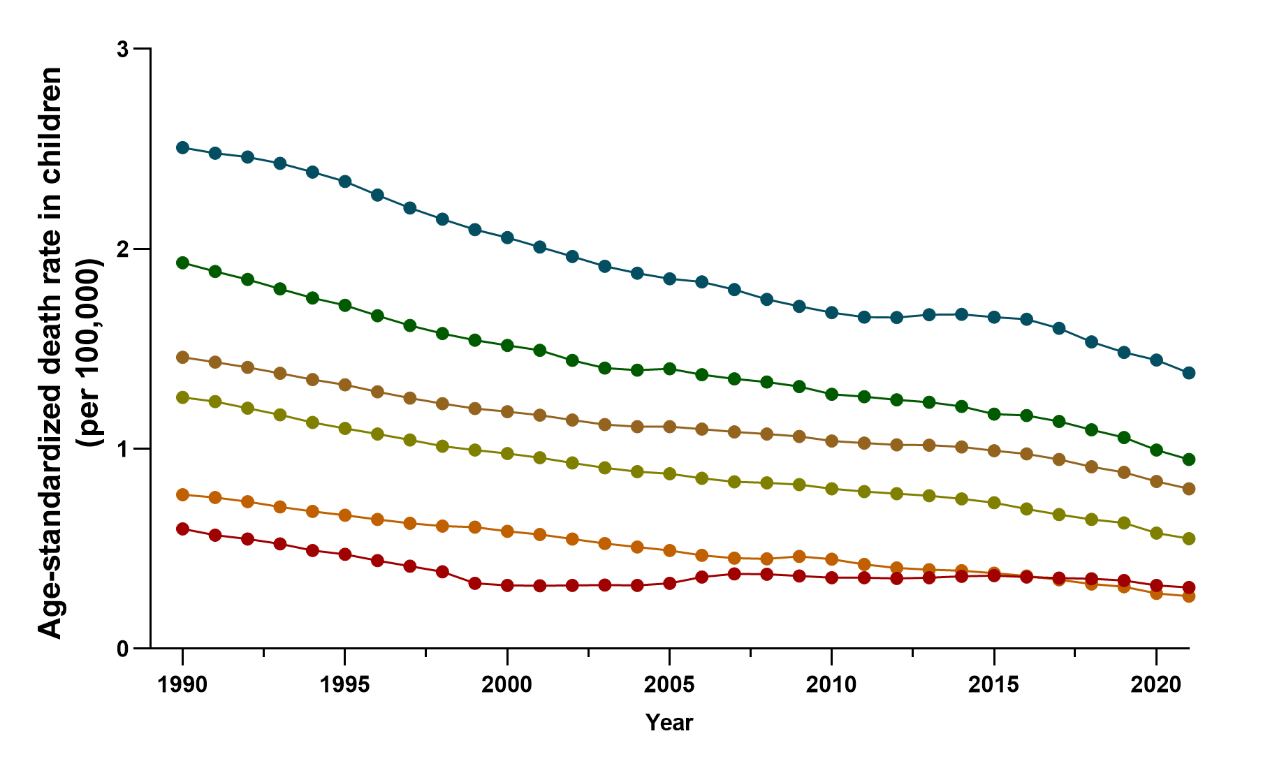

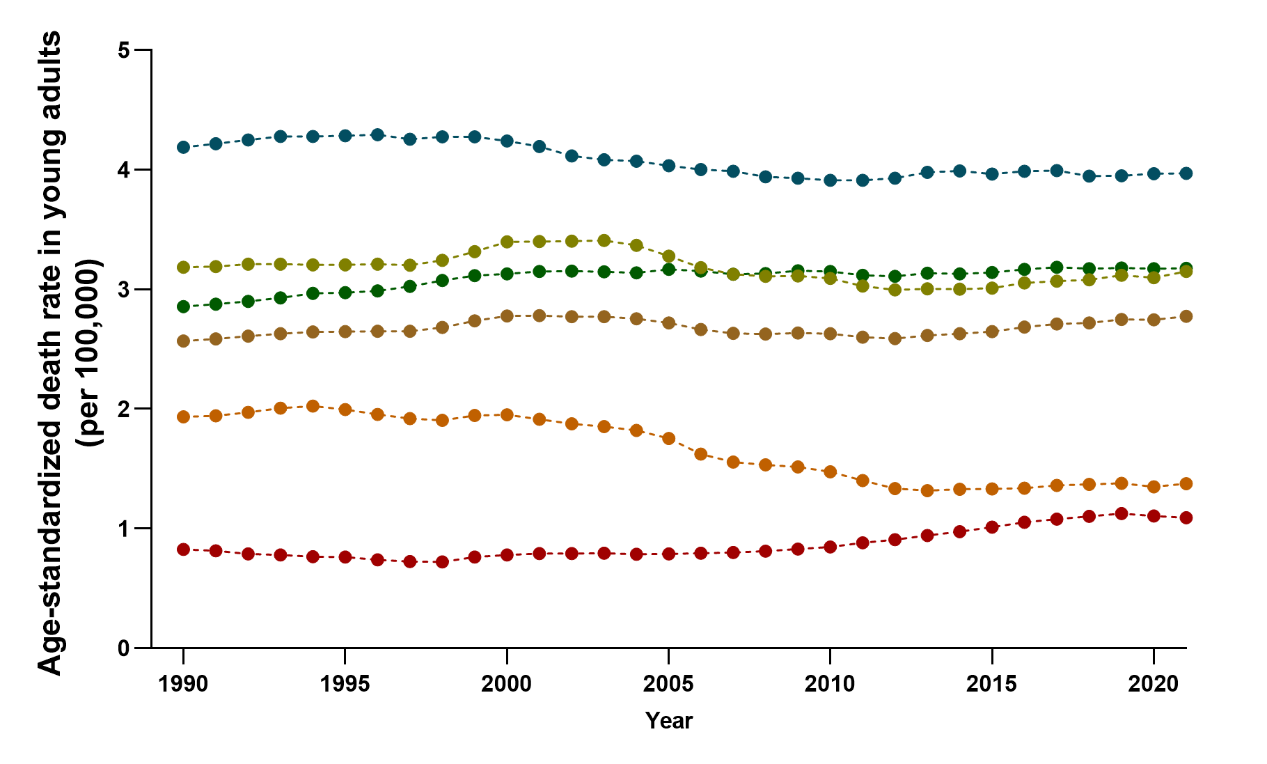


**Supplemental figure 1 Chronic kidney diseases related death rate from 1990 to 2021 in children and young adults.**


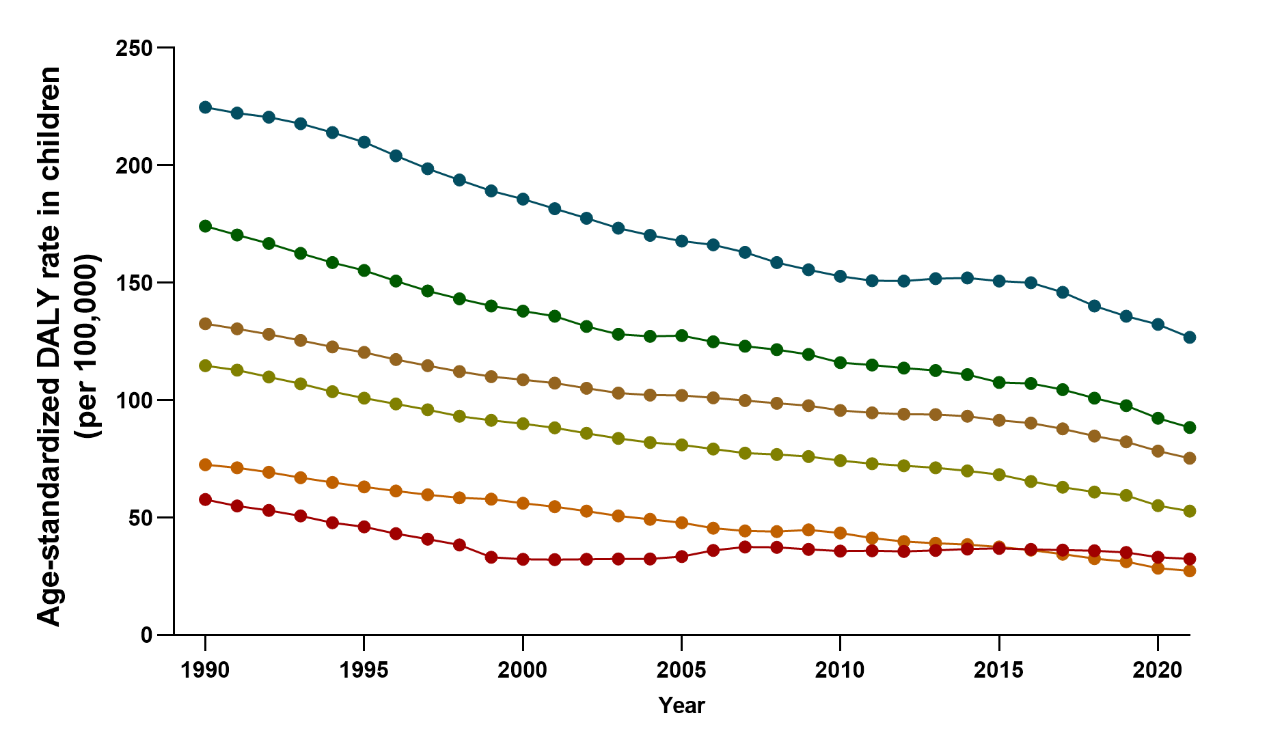

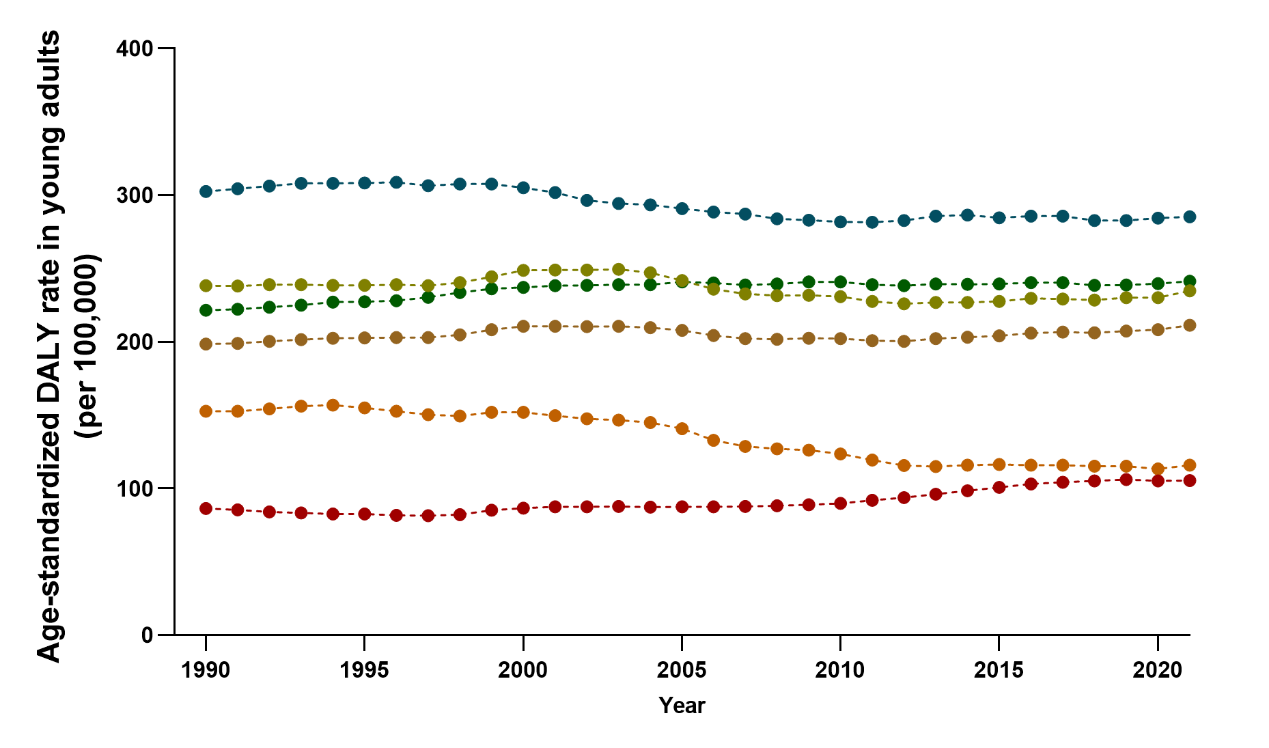


**Supplemental figure 2 Chronic kidney diseases related DALY rate from 1990 to 2021 in children and young adults.**


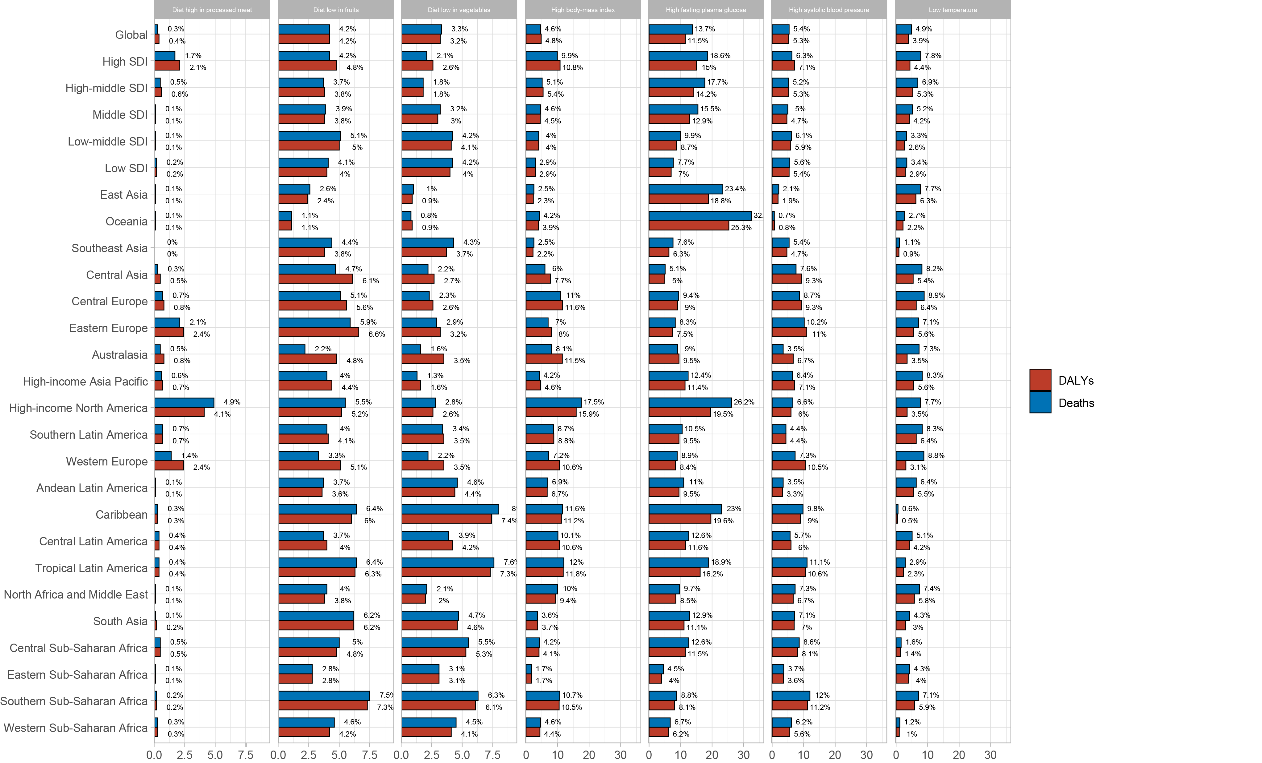

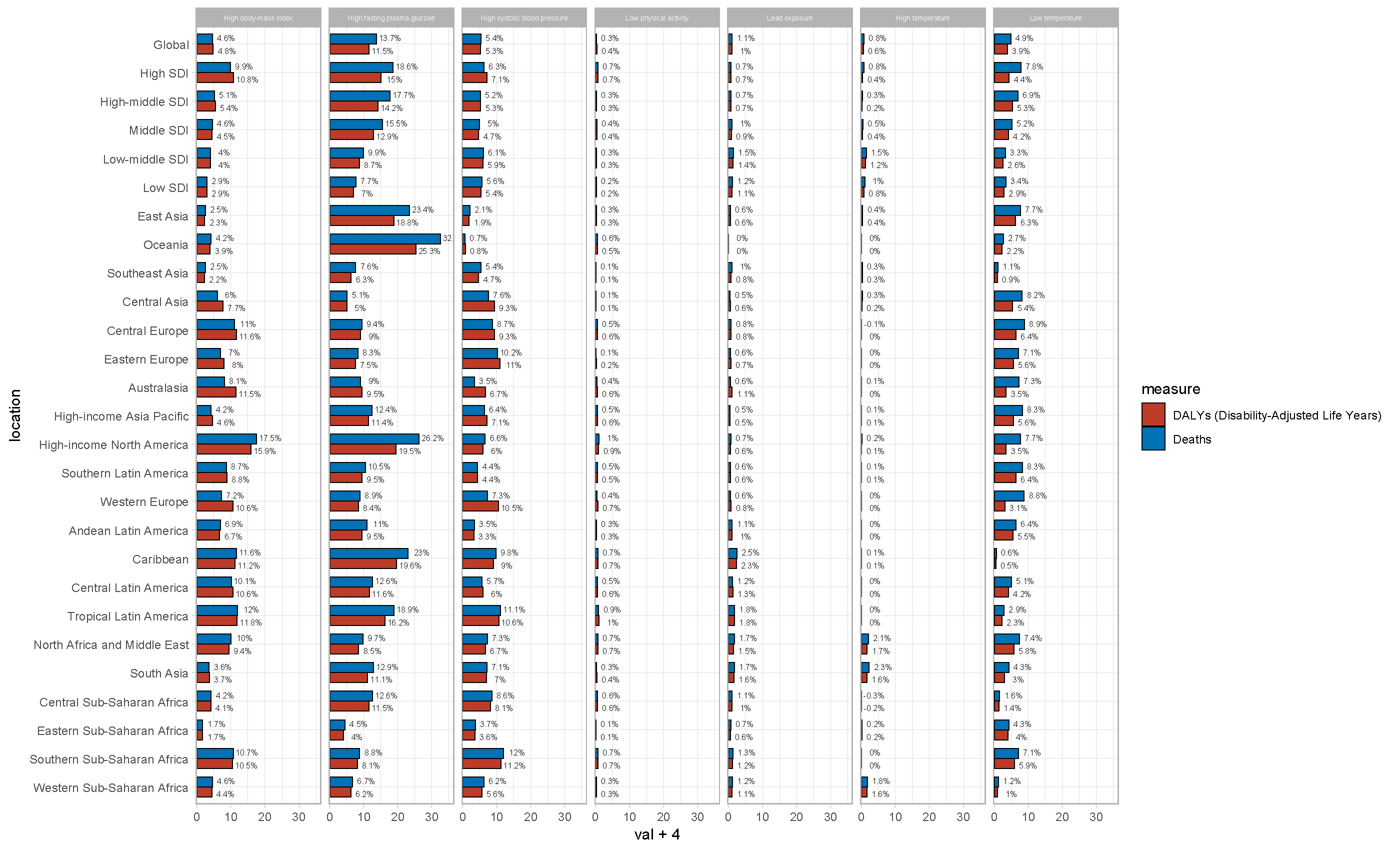


**Supplemental figure 3 The burden of chronic kidney diseases attributable to risk factors in 1990 in young adults.**


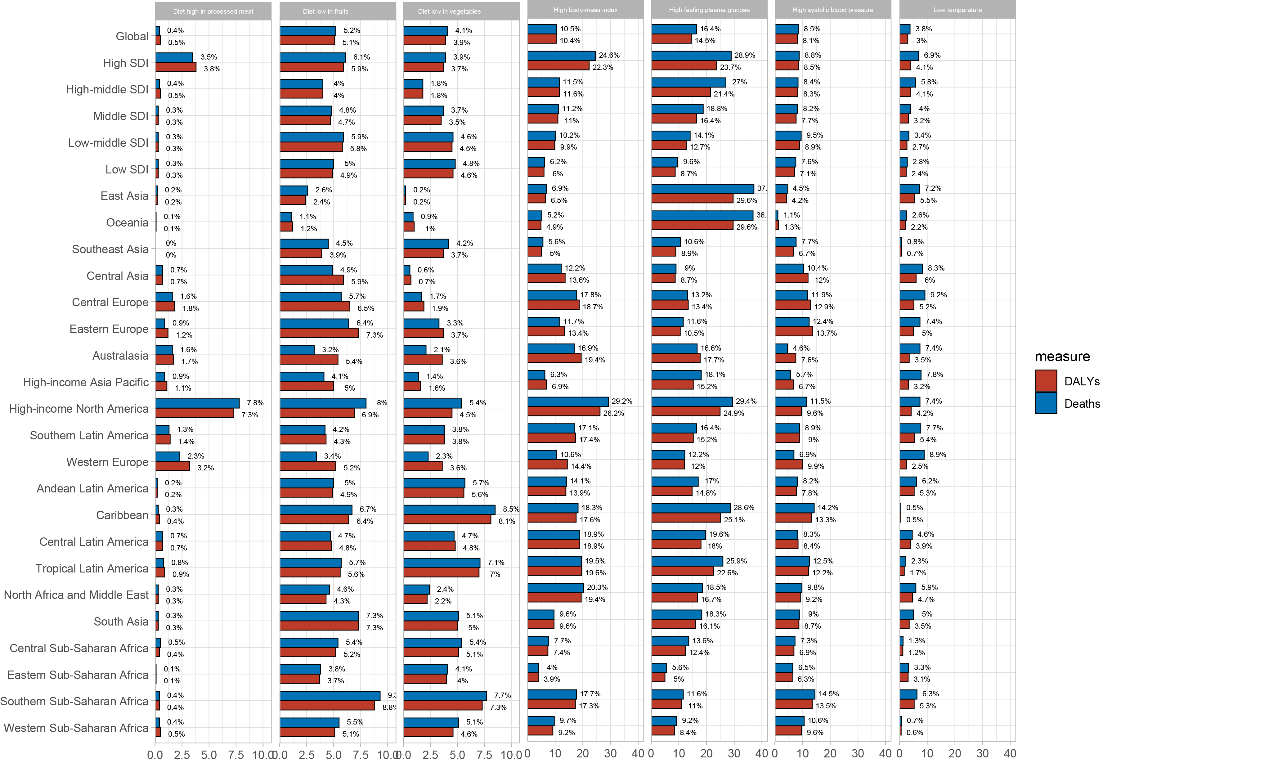

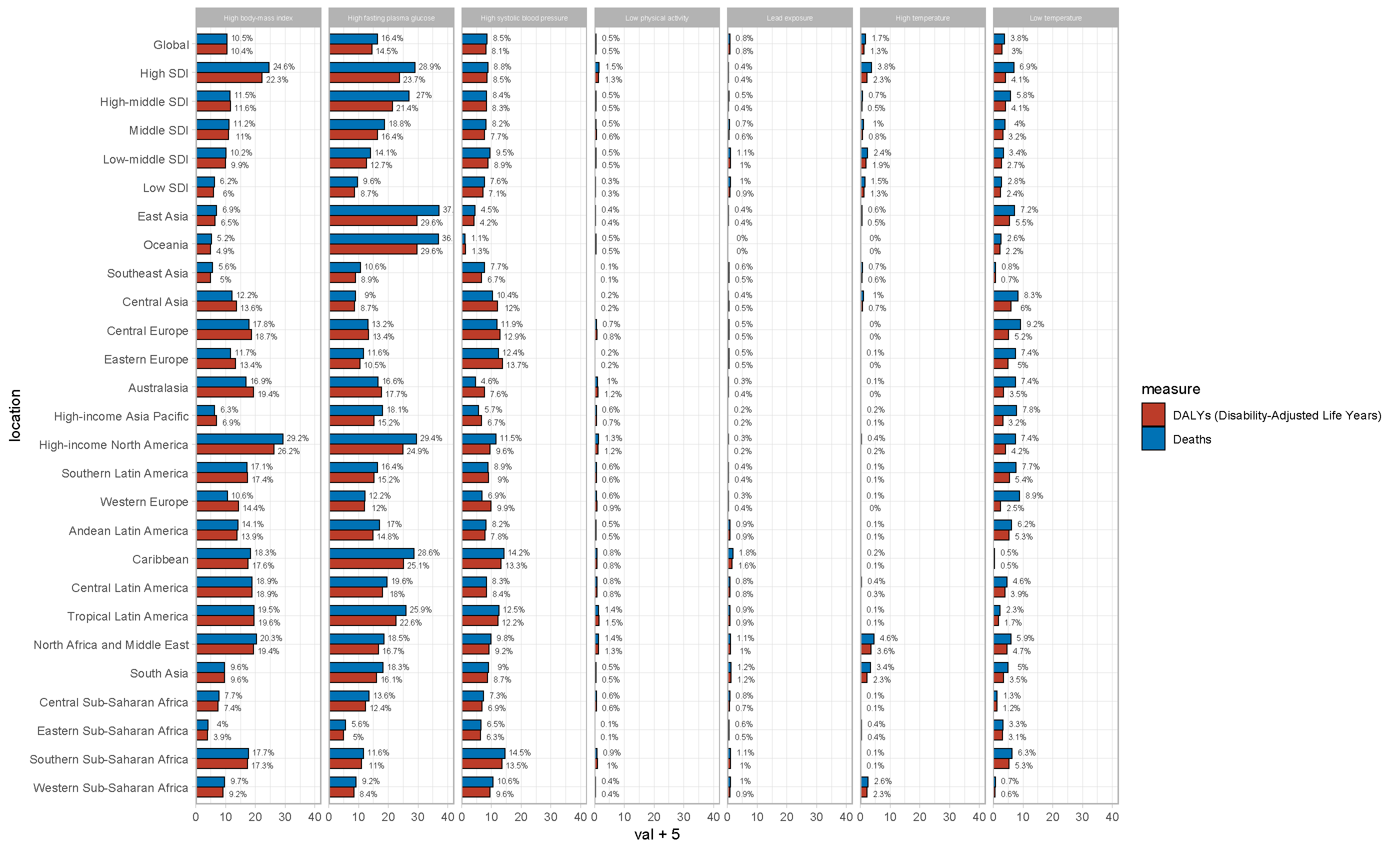


**Supplemental figure 4 The burden of chronic kidney diseases attributable to risk factors in 2021 in young adults.**


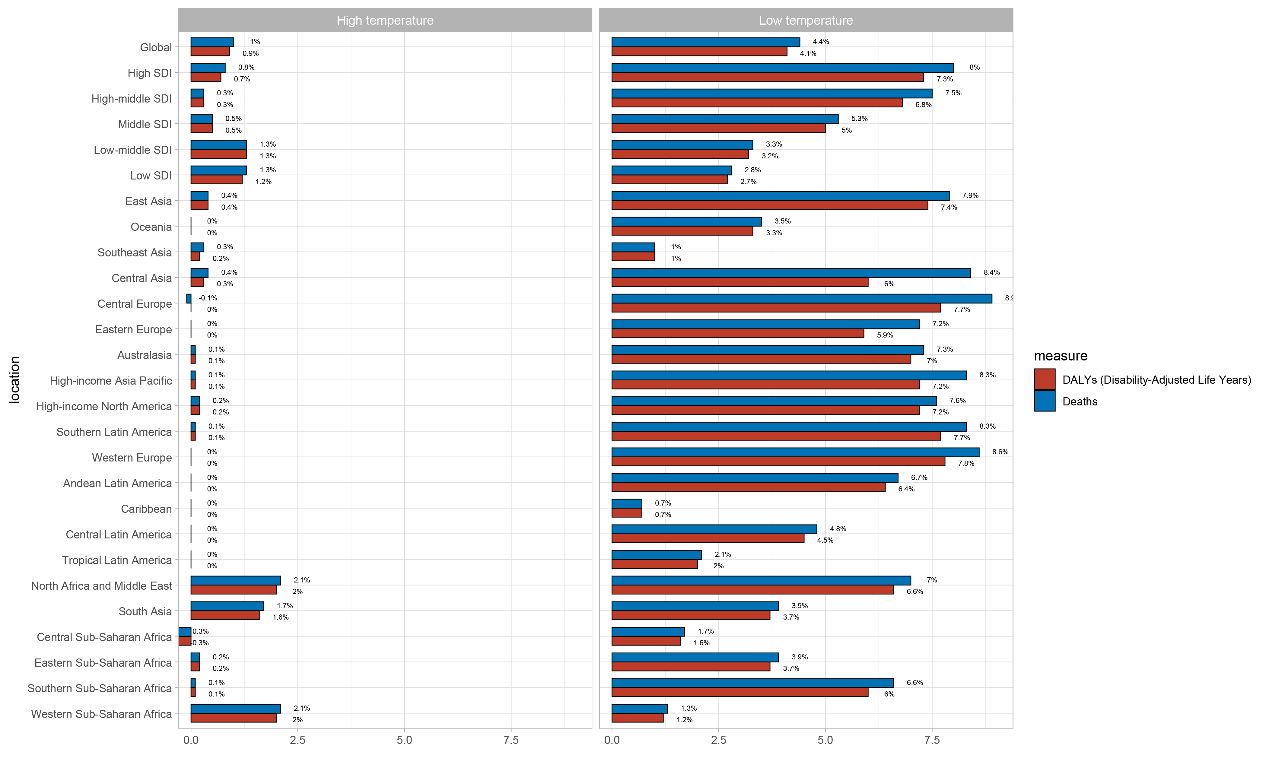

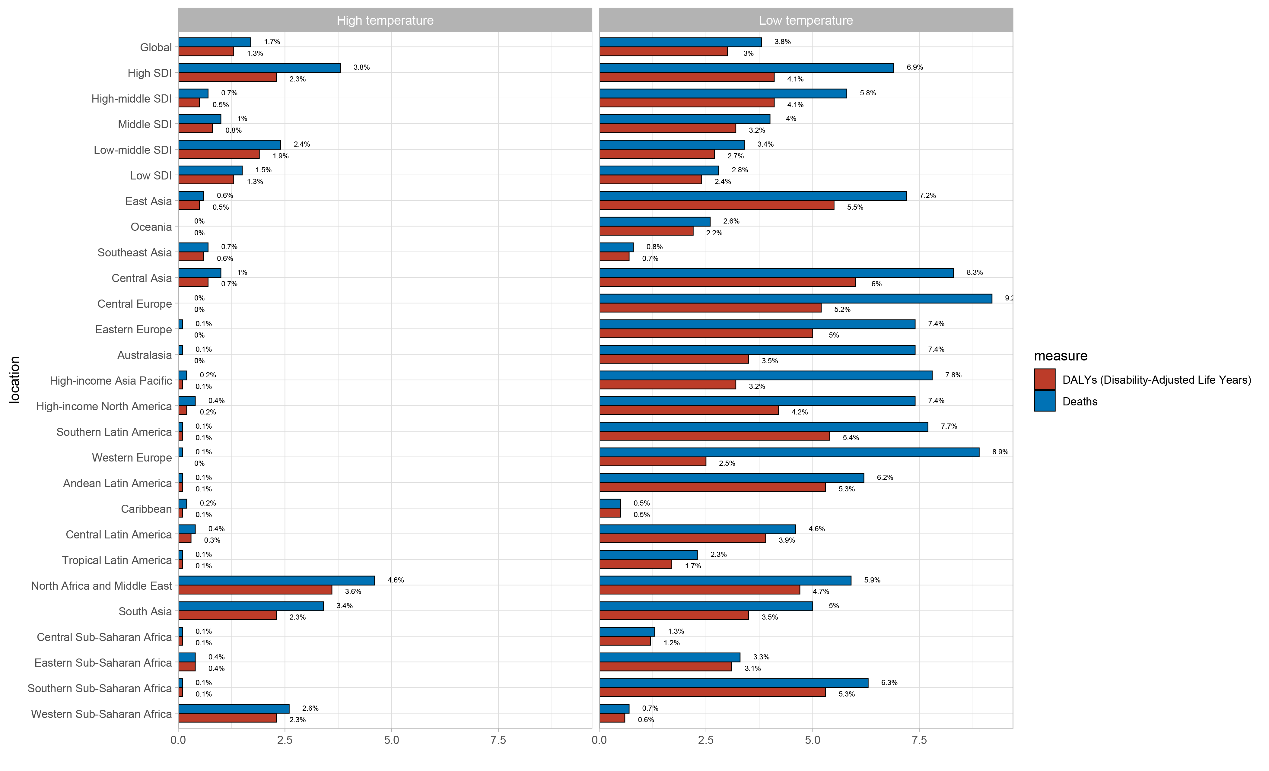


**Supplemental figure 5 The burden of chronic kidney diseases attributable to risk factors from 1990 to 2021 in children.**

**
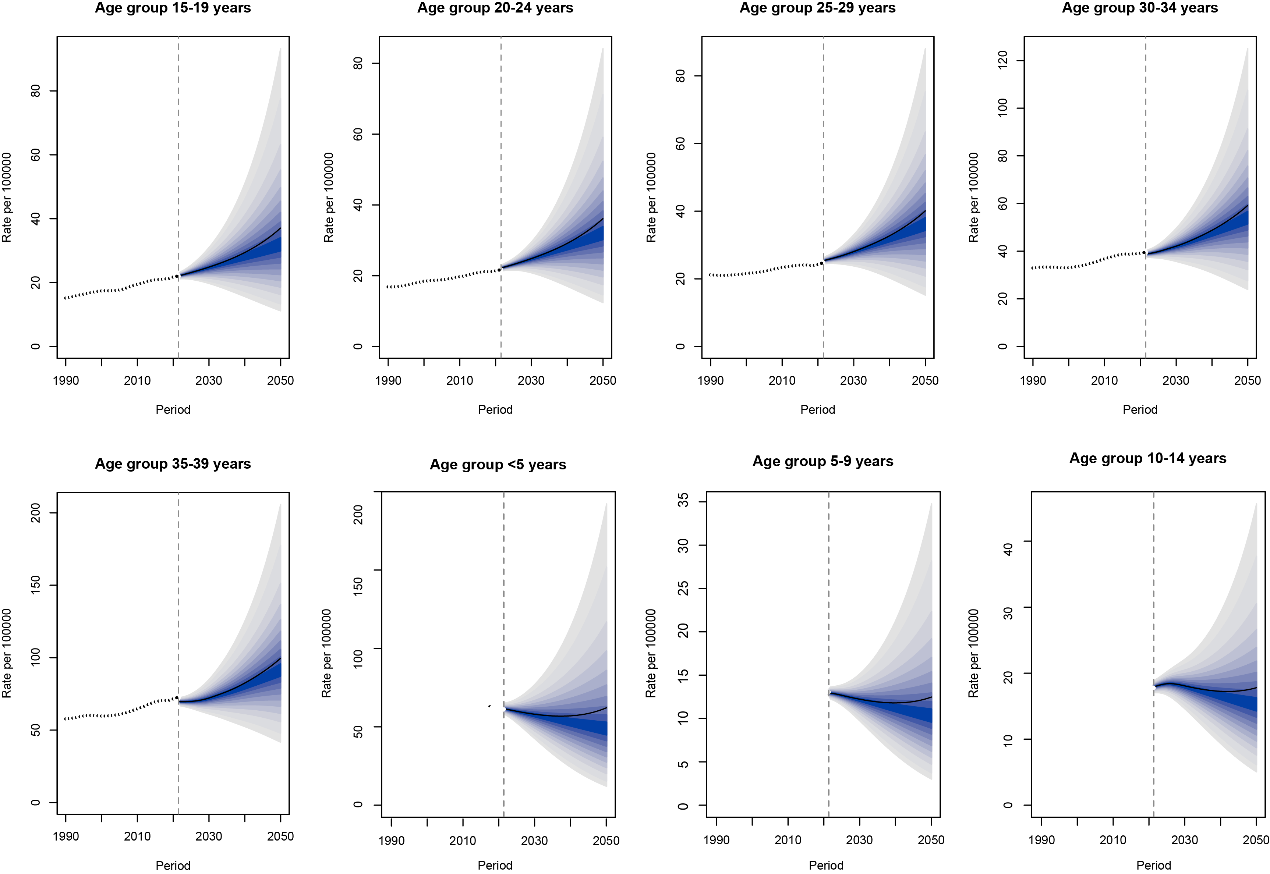
Supplemental figure 6 Future forecast of chronic kidney disease related incidence rate in different age groups. The dotted line indicates 2021, the actual incidence on the left side of the dotted line, and the predicted incidence on the right side of the dotted line.** **The solid black line in dark blue is the predicted trend of incidence.**
